# The effect of cost-sharing design characteristics on use of health care recommended by the treating physician; a discrete choice experiment

**DOI:** 10.1186/s12913-018-3598-4

**Published:** 2018-10-20

**Authors:** Benjamin H. Salampessy, Maaike M. Alblas, France R. M. Portrait, Xander Koolman, Eric J. E. van der Hijden

**Affiliations:** 1 Department of Health Sciences, Faculty of Science, Vrije Universiteit Amsterdam, De Boelelaan 1085, 1081 HV Amsterdam, The Netherlands; 2 Department of Public Health, Erasmus Medical Centre - University Medical Center Rotterdam, P.O. 2040, 3000 CA Rotterdam, The Netherlands

**Keywords:** Cost-sharing, Price transparency, Discrete choice experiment, Health reform, Managed care

## Abstract

**Background:**

Cost-sharing programs are often too complex to be easily understood by the average insured individual. Consequently, it is often difficult to determine the amount of expenses in advance. This may preclude well-informed decisions of insured individuals to adhere to medical treatment advised by the treating physician. Preliminary research has showed that the uncertainty in these cost-sharing payments are affected by four design characteristics, i.e. 1) type of payments (copayments, coinsurances or deductibles), 2) rate of payments, 3) annual caps on cost-sharing and 4) moment that these payments must be made (directly at point of care or billed afterwards by the insurer).

**Methods:**

An online discrete choice experiment was used to assess the extent to which design characteristics of cost-sharing programs affect the decision of individuals to adhere to recommended care (prescribed medications, ordered diagnostic tests and referrals to medical specialist care). Analyses were performed using mixed multinomial logits.

**Results:**

The questionnaire was completed by 7921 members of a patient organization. Analyses showed that 1) cost-sharing programs that offer clear information in advance on actual expenses that are billed afterwards, stimulate adherence to care recommended by the treating physician; 2) the relative importance of the design characteristics differed between respondents who reported to have forgone health care due to cost-sharing and those who did not; 3) price-awareness among respondents was limited; 4) the utility derived from attributes and respondents’ characteristics were positively correlated; 5) an optimized cost-sharing program revealed an adherence of more than 72.9% among those who reported to have forgone health care.

**Conclusions:**

The analyses revealed that less complex cost-sharing programs stimulate adherence to recommended care. If these programs are redesigned accordingly, individuals who had reported to have forgone a health service recommended by their treating physician due to cost-sharing, would be more likely to use this service. Such redesigned programs provide a policy option to reduce adverse health effects of cost-sharing in these groups. Considering the upcoming shift from volume-based to value-based health care provision, insights into the characteristics of a cost-sharing program that stimulates the use of recommended care may help to design value-based insurance plans.

**Electronic supplementary material:**

The online version of this article (10.1186/s12913-018-3598-4) contains supplementary material, which is available to authorized users.

## Background

Policy makers around the world have introduced cost-sharing programs to control rising health care expenditure. These programs introduce financial incentives for insured individuals to increase their awareness of health care costs. Increased cost-awareness is believed to lead to a well-informed decision to use health care, where expected medical benefits and costs have been considered [1]. Numerous studies have shown that cost-sharing reduces the demand of care [1, 2]. The RAND Health Insurance Experiment (HIE) showed that higher cost-sharing leads to a decrease in demand of care with no effect on health except for those with the lowest income and poor initial health. The HIE also showed that cost-sharing reduces utilization of both care ‘*recommended’* by physicians and care that was ‘*non-recommended’* [3]. Recommended care consists of health services that have relatively high medical benefits as assessed by physicians, whereas non-recommended care is judged by physicians to have relatively little to no medical benefit [3]. In many European countries (e.g. the Netherlands, UK, Denmark) and in some US health plans, General Practitioners (GPs) act as gatekeepers for the more costly specialist care [4, 5]. As gatekeepers, GPs assess the necessity of care at the individuals’ first contact with health care and decide whether to refer them. GP care may also be exempt from cost-sharing to ensure initial care-seeking decision, but the referred (recommended) care is generally subject to cost-sharing [6, 7]. However, it is up to individuals to decide (i.e. individuals’ choice) to follow up on recommended care. In literature, costs have been described as possible reason for not using recommended care [8, 9]. It remains unclear which characteristics of a cost-sharing program stimulate the decision of individuals to follow up on recommended care and to what extent.

A common problem with cost-sharing programs is that they are too complex to be easily understood by the average insured individual [6, 10]. The level of complexity makes it often difficult to determine the amount of expenses in advance and may preclude well-informed decisions. For example, providers are often unable to inform the individual on the combination of treatments and prices in advance, and cost-sharing may only be applicable for specific health services, it may vary across health plans and some groups may be exempt from these payments. Therefore, in many instances, insured individuals only know the final amount they need to pay when they actually receive their bill. Moreover, some individuals are more able to comprehend complex health information than others. This factor is referred as health literacy, i.e. the ability to process and understand basic information needed to make appropriate health decisions [11]. Given that limited health literacy is more concentrated in specific subgroups of the population (e.g. individuals in poor health, with low socio-economic status) [12], these individuals are therefore less able to determine the amount of their expenses in advance. Uncertainty over expenses may cause some individuals not to use health care they would have used had they known these expenses in advance. Therefore, this study aims at understanding which characteristics of a cost-sharing program reduce its complexity in order to improve adherence to recommended care.

### Cost-sharing design characteristics

We have examined a number of cost-sharing programs from the perspective of an insured individual to assess which characteristics contribute to their complexity. This involved a review of the literature provided and expert consultations (a summary is provided in Additional file 1). Our research has showed that, in general, the complexity of cost-sharing programs is primarily affected by two design choices made by policy makers: the actual amount of cost-sharing payments and the moment these payments must be paid.

With respect to the first design choice, there are three key characteristics that determine these amounts. The first characteristic, *type of cost-sharing payments* (i.e. copayments, coinsurances and deductibles), affects how the amount of cost-sharing is calculated. Copayments consist of flat-rate fees per unit of care that result in fixed fees known by insured individuals in advance. In contrast, for coinsurances (fees equal to a percentage of the price per unit of care) and for deductibles (insured individuals bear costs up to an annual fixed amount while the health insurer (HI) covers all exceeding costs) the actual amount depends on actual prices of health services. Given that price transparency is generally limited [13], it is difficult for insured individuals to determine the exact amount of payments in advance. Hence, coinsurances and deductibles contribute to uncertainty over the actual amount of cost-sharing payments.

The second characteristic is the *rate of cost-sharing payments* since levels vary across different health services and even within a given service. For example, rates for prescribed medication are generally lower than those for visits to a medical specialist. Moreover, different tiers may be applied to take the effectiveness of the prescribed medication into account. Individuals pay a lower rate if their medication is considered evidence-based (e.g. insulin for diabetes patients) [14].

The third characteristic, *annual caps on cost-sharing*, relates to protection mechanisms generally built into cost-sharing programs which limit the actual amount of cost-sharing payments an individual will have to make per year. These caps may vary across specific groups (e.g. older individuals), health services (e.g. separate caps for hospital stays and medications) or income (e.g. low-income groups). Additionally, some groups may be entitled to multiple caps simultaneously. Given the administrative process required for billing cost-sharing payments, individuals may not always have an up-to-date overview of applicable caps. Furthermore, not all individuals are fully aware of all the caps applicable to their individual situation. Hence, the complexity in the design of annual caps also increases uncertainty concerning the amount of cost-sharing payments.

The second design choice relates to the *moment* that cost-sharing payments must be paid and reflects a fourth design characteristic of cost-sharing programs. Insured individuals may pay their cost-sharing bill directly at the point of care, namely where the health services are actually provided or bought (e.g. when picking up medication at a pharmacy). Alternatively, the entire bill is sent to a third-party payer (e.g. HI) who bills individuals afterwards for their cost-sharing payments only. Given that the administrative process may take several weeks or even months to complete, this introduces uncertainty as to the moment that cost-sharing payments need to be paid.

### Policy choices in the design of cost-sharing programs

Most cost-sharing programs in developed countries involve combinations of the four aforementioned design characteristics. For example, in the US, copayments are generally paid for prescribed medication and this is combined with different tiers to encourage individuals to use lower-cost alternatives. Annual out-of-pocket costs are limited to $3500 per person or $9400 per family while specific health services (e.g. prenatal and postnatal care) are exempt from cost-sharing [15]. France applies fixed copayments for prescribed medication (€0.50 per drug), a combination of coinsurance (20%) and copayments (€16–20) for inpatient hospital services and again a combination for a consult of the GP or specialist [5].

Current cost-sharing programs have been designed in the context of Fee-For-Service as dominant provider payment model. Considering the upcoming shift from volume-based to value-based health care provision, new payment models are increasingly used [16]. For example, bundled payments are used to pay one overall price for the full cycle of care that may cover health services of multiple providers. Cost-sharing programs may be designed to stimulate the use of in-bundle care rather than to reduce moral hazard [17]. Hence, insights into the characteristics of a cost-sharing program that stimulates recommended care may help to design value-based insurance plans.

### Our study

In order to examine the effect of less complex cost-sharing programs on the adherence to recommended care, we conducted a discrete choice experiment (DCE) to estimate preferences with respect to the four cost-sharing design characteristics. In the experimental settings of a DCE, respondents evaluate hypothetical, but realistic cost-sharing programs based on the four design characteristics. Although the programs are per definition hypothetical, we tried to reflect real-world decision making of respondents in the DCE and focused on health services covered by the Dutch mandatory basic health insurance, ordered or prescribed by the treating physician (*recommended care*) and subject to a deductible (€385 in 2016) that is billed afterwards by the HI.

To recap, the purpose of this study is to assess to what extent four design characteristics (namely the type and rate of cost-sharing payments, annual caps and the moment that these payments are paid) affect the decision to utilize (i.e. follow up on) recommended care by the treating physician, and whether this response to cost-sharing differs across individuals.

## Methods

To answer our research question, we conducted a DCE among patients who have received follow-up care recommended by their treating physician and had to decide whether or not to follow up on this referral. In this survey technique, respondents are presented with several hypothetical scenarios (choice sets) consisting of two or more alternatives (i.e. cost-sharing programs) that systematically vary the attributes (i.e. the four design characteristics) across given levels. Respondents are asked to choose their most preferred alternative in each choice set. In accordance with economic theory [18, 19], the chosen alternatives are considered to be those which yield the highest utility and reflect the respondent’s latent preferences.

In the DCE, we focused on three types of health services recommended by the treating physician (i.e. prescribed medications, ordered diagnostic tests and referrals to medical specialists for consultation or treatment (specialist care)) and subject to cost-sharing, specifically deductibles. As stated, we did not focus on the initial decision of insured individuals to visit their GP exempted from cost-sharing, but focused on their decision to follow up on care recommended by the treating physician and subject to cost-sharing payments.

### Attributes and levels

The selection of attributes was based on preliminary research as described in the background section. Hence, the four attributes (one for each design characteristic) were included in the DCE (shown in Table 1). We aimed to keep the levels of the second attribute (*rate of payments*) independent from the first attribute (type of payments) and used one general price per type of health service to determine levels (€9.50 for medication; €250 for diagnostic tests; €2000 for specialist care). For example, the reference level in specialist care reflected a rate of payment equal to €200 in both copayments and coinsurances (10%*€2000 = €200) payments. A few levels were rounded to the nearest whole number to reduce the cognitive burden of the DCE (e.g. for diagnostic tests, the reference level in case of coinsurances (€80/€250 = 32%) was rounded to 30%). For medication, the lowest rate of copayments was assigned based on feedback of experts as they had perceived the difference of €1 with the subsequent level to be too little and unrealistic.

**Table 1:** Attributes and levels per type of health service

Attributes	Types of health service		
	Medication	Diagnostic tests	Specialist care
1) Type of payments			
*Type1*	Copayments		
*Type2 (reference)*	Coinsurances		
2) Rate of payments			
*Rate1*	€2 / 40%	€40 / 15%	€100 / 5%
*Rate2*	€4 / 50%	€50 / 20%	€140 / 7%
*Rate3 (reference)*	€7 / 70%	€80 / 30%	€200 / 10%
3) Annual caps on cost-sharing			
*Cap1*	Half cap+		
*Cap2 (reference)*	Full cap+		
4) Moment of payment			
*Moment1*	Afterwards billed by health insurer		
*Moment2 (reference)*	Directly at point of care		

+The situation with a full remaining annual cap (full cap) reflected a situation with no previously made payments while in the other situation (half cap) payments had been previously made equal to half of the annual cap. The full cap was set equal to the current Dutch deductible (in 2016, €385)

### Experimental design

Given that annual caps are fixed for a whole year, varying caps within a choice set was not considered realistic. Instead, this attribute only varied across choice sets. To achieve this, we used only the first (*type of payments*), second (*rate of payments*) and fourth (*moment of payment*) attribute to generate a D-efficient design (main-effects, zero priors) blocked into four blocks (three choice sets per block) and included this design twice in questionnaire to incorporate the third attribute (*annual caps on cost-sharing*). By doing so, for each type of health service two blocks were included in the questionnaire, i.e. three choice sets with ‘half cap’ and three choice sets with ‘full cap’. Each choice set consisted of two unlabeled hypothetical cost-sharing programs and an opt-out (i.e. the option to forgo health care without having to make any payments) of which the respondents were asked to choose their most preferred option. The opt-out was included to better reflect real-life settings in which insured individuals may choose not to use health care [20]. To optimize the validity and reliability of our results, we wanted to remain as close as possible to the real-world decision making of respondents. Therefore, respondents were instructed to use the most recent situation as context situation in the DCE in which they had visited their treating physician and received a follow-up for one of the health services (medication, diagnostic tests or specialist care) subject to cost-sharing payments, specifically deductibles payments, within the last 2 years, and had forgone the given service due to these payments (group: *forgoing health care*). If respondents had not forgone these health services during this period, they were instructed to use the most recent situation in which they had used the aforementioned health services recommended by the treating physician (group: *utilizing health care*). Therefore, respondents completed 0, 6, 12 or 18 choice sets depending on whether they had been recommended the given health service, i.e. either have been used or forgone prescribed medication, ordered diagnostic tests or referred specialist care in the last 2 years.

### Questionnaire

In addition to the DCE, the questionnaire contained questions concerning the deductibles paid by the respondent, respondents’ personal and socio-economic characteristics, health and sense of mastery, and open-ended feedback questions (the full questionnaire is provided in Additional file 2). Sense of mastery refers to the extent to which individuals perceive life events (e.g. the development of chronic conditions) as being under their control, as opposed to being determined by external factors [21]. Sense of mastery was included because it has been shown to modify the impact of these events on health [22]. The questionnaire was piloted online using a small sample of the study population (*n* = 269, not included in analyses) resulting in minor adjustments in wording but no changes in attributes, levels or experimental design. To estimate the minimum sample size (main effects), we used Johnson and Orme’s rule-of-thumb [23, 24]. The minimum number of respondents was estimated to be 84 per block.

### Study population

We selected the online panel of the Dutch Patient Federation. Panel members are frequent users of health care and are more likely to face cost-sharing payments making them highly relevant for our study. In March and April 2016, all panel members (*n* = 23,394) were asked by email to complete the online questionnaire on a voluntary basis.

### Data analysis

Data was analyzed separately per group (respondents reported having forgone health care and those who did not) for each type of health service. In total, six analyses were performed using mixed multinomial logits (MMNL) to account for the clustering of data (i.e. the same respondent completed multiple choice sets) and to account for random taste in preferences [20]. Each MMNL modelled the following equation:

1 $$ {V}_{ij}={\beta}_1\ast Type1+{\upbeta}_2\ast Rate1+{\upbeta}_3\ast Rate2+{\upbeta}_4\ast Cap1+{\upbeta}_5\ast Cap1\ast Type1+{\upbeta}_6\ast Cap1\ast Rate1+{\upbeta}_7\ast Cap1\ast Rate2+{\upbeta}_8\ast Cap1\ast Mo\mathrm{m} ent1+{\upbeta}_9\ast Mo ment1+{\upbeta}_{10}\ast Alternative\ Specific\ Constant\ (optout)+{\upvarepsilon}_{\mathrm{ij}} $$

In Eq. 1, the utility that individual *i* derived from a hypothetical cost-sharing scheme alternative in choice set *j* is reflected by *V*_*ij*_ and characterized by the combination of different levels of attributes (β_1_-β_4,_ β_9_). Labels of the included variables correspond with the attributes’ labels used in Table 1. Given its fixed nature, the third attribute (*annual caps on cost-sharing)* was interacted (β_5_-β_8_) with the other attributes. An alternative specific constant was also included (β_10_) to reflect the different nature of the opt-out option and the average derived unobserved utility. Given its coding (1 if the opt-out option was chosen and 0 otherwise), a positive sign indicates a general preference to forgo health care in any cost-sharing scheme while a negative sign indicates a general preference to utilize health care in similar programs. The error term (ε) was assumed to be normally distributed. Effects coding was used for all attributes. Coefficients for the omitted levels were calculated as the negative sum of the estimated coefficients [25].

To make our findings representative to the Dutch general population, we repeated our main analyses using inverse probability weighting (IPW) based on age, gender, educational level and EQ5D scores [26]. Weights were computed using iterative proportional fitting (also referred as ‘raking’) such that the weighted marginal totals of our sample closely matched those of the total population [27, 28].

Additional analyses were performed to identify differences in preferences across subgroups by using interaction terms between attributes and respondents’ characteristics (i.e. subjective health status, income, financial situation, sense of mastery and educational level). In interest of brevity, the modelled MMNLs are described in Additional file 3.

To assess the effect of an ‘*optimally’* designed cost-sharing program, we used sample enumeration to calculate average choice probabilities separately for both groups per type of health service. These probabilities reflect the expected share of respondents that would choose the service if cost-sharing programs were designed according to the respondents’ preferences [20]. First, we calculated utility scores for an alternative to use the health service in an optimally designed program, and an alternative to forgo this service (opt-out option). The alternative yielding the highest utility score was assumed to be chosen by the respondent. Since random parameters were included, choice probabilities could not be calculated directly. Hence, a random sample of 5000 respondents was simulated and corresponding probabilities were calculated using 1/(1 + exp.^-V^) in which V reflects the utility derived from ‘*optimally’* designed cost-sharing program [20]. Subsequently, the average choice probability was calculated by taking the average of all simulated probabilities.

Experimental designs were generated in SAS/STAT software version 9.3 [29]. In the final data analyses, random parameters assumed a normal distribution and estimated based on 5000 Halton draws. MMNL models were estimated in Stata 14.1 [30]. Model fit was assessed based on -2log likelihood functions. Results were considered statistically significant if *p*-values ≤.05.

## Results

### Study population

The questionnaire was completed by 8734 respondents (response rate 37.3%) of whom 813 respondents were excluded for various reasons described in Additional file 4. Of the 7921 included respondents (Table 2), 1048 respondents (13.2%) reported having forgone health care recommended by their treating physician due to cost-sharing payments (group*: forgoing health care*). These respondents were, on average, significantly lower educated and had a lower net income, a worse financial situation, a lower level of health and mastery compared to those who had not forgone health care recommended by their treating physician due to cost-sharing payments (group: *utilizing health care*).

**Table 2:** Characteristics of 7921 respondents included in the study

		Total	Forgoing health care (*n* = 1048)	Utilizing health care (*n* = 6873)
Demographics				
Age (in years)^**^	Mean (sd)	62.3 (11.1)	57.8 (10.9)	63.0 (11.0)
Gender (%)^**^	Men	47.9	39.4	49.2
Socio-economic status				
Education level (%)^**^	Low	24.9	27.5	24.6
	Moderate	32.0	35.6	31.4
	High	43.1	36.9	44.0
Monthly net income (%)^**^	< €1000	8.4	19.5	6.7
	€1001–€2000	30.9	43.4	29.0
	€2001–€3000	24.4	15.7	25.7
	€3001–€4000	12.1	5.0	13.2
	>€4000€	6.7	2.1	7.4
	Unknown or not-disclosed	17.5	14.3	18.0
Financial situation (%)^**^	Running up debts or	6.7	20.4	4.7
	Using savings	16.6	24.3	15.4
	Precisely enough to live from	33.7	36.5	33.2
	Saving a small amount	37.0	16.5	40.2
	Saving a large amount	6.0	2.3	6.5
Health				
Subjective health (%)^**^	Very poor	2.8	3.6	2.7
	Poor	16.1	18.1	15.7
	Moderate	41.7	46.2	41.1
	Good	35.3	27.8	36.4
	Very good	4.1	4.3	4.1
Chronic conditions (%)^*^	One or more	84.0	81.6	84.3
Health Status, EQ-5D-5 L^**^	Mean (sd)	.76 (.20)	.71 (.23)	.77 (.20)
Sense of mastery, Pearlin ^**^	Mean (sd)	22.3 (5.8)	20.5 (5.9)	22.6 (5.7)
Respondents per health service				
Number (% of group)+	*Medication*		475 (45.3%)	6277 (91.3%)
	*Diagnostic tests*		738 (70.4%)	5510 (80.2%)
	*Specialist care*		662 (63.2%)	4702 (68.4%)

Respondents were classified as divided in two groups; respondents reported having forgone health care due to deductibles (group: forgoing health care) and those did not (group: utilizing health care). Health status was measured by the Euroqol-5D-5 L questionnaire and valued according to Dutch tariffs. Scores range from 0 (a health status equal to death) to 1 (a health status equal to perfect health). The sense of mastery was measured by the Pearlin Mastery Scale Test. Seven statements (e.g. “I have little control of events that happen to me”) are scored on a five point Likert scale. Summed scores range from the lowest possible score of 7 (lacking sense of mastery) to the highest possible score of 35 (complete sense of mastery) [21]

** *p*-value ≤.01, * *p*-value ≤.05, +as percentage of group forgoing health care (*n* = 1048) and utilizing health care (*n* = 6873) respectively, sd: standard deviation

### Preferences

**Fig. 1:**
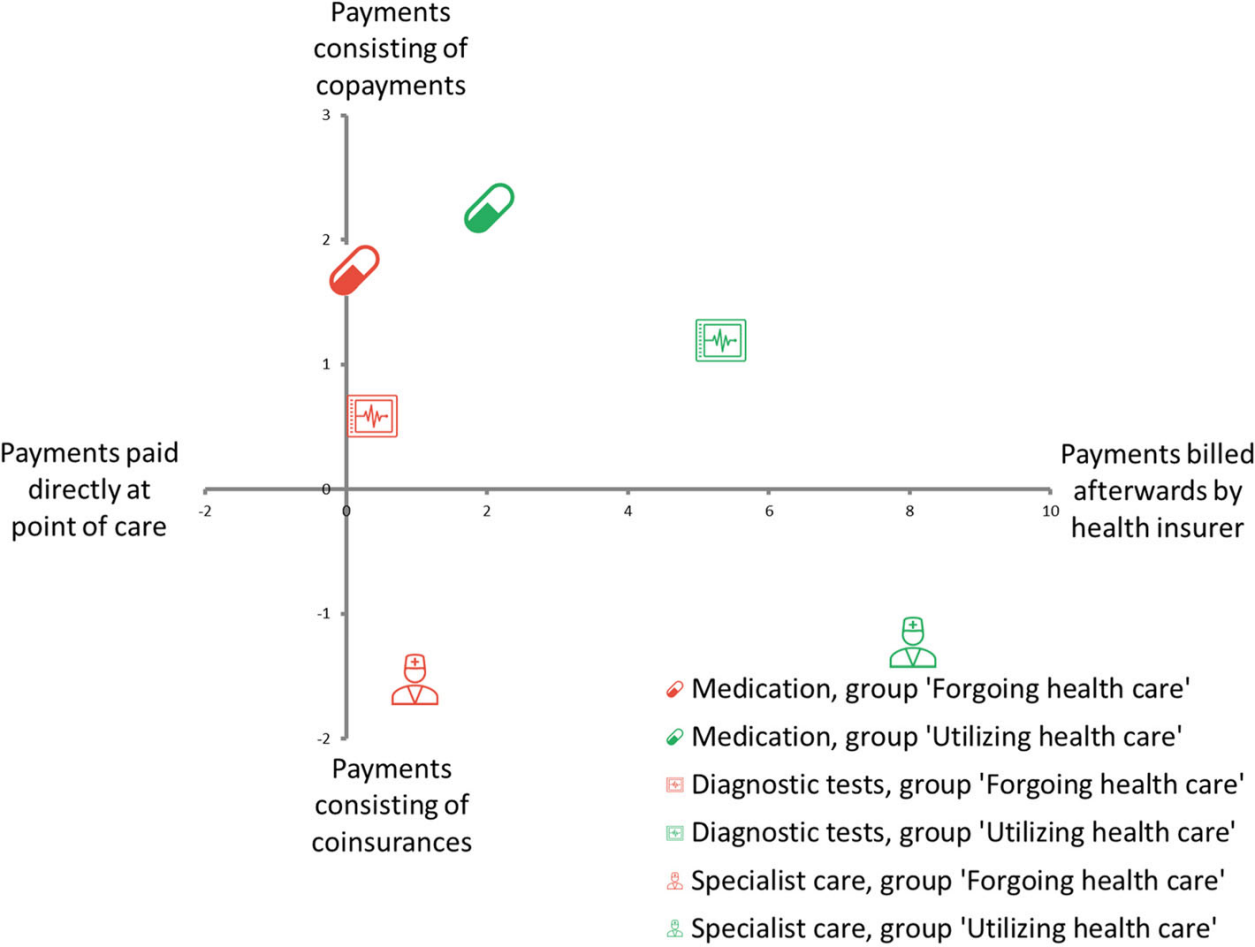
Ratio of derived utility moment of payment and type of payments, standardized by rate of payments. Coefficients of the attributes *moment of payments* and *type of payments* have been standardized by dividing the utility derived of each attribute by the utility derived from the most preferred level of the attribute *rate of payments*, i.e. rate1. In only one analysis (for medication, group ‘Forgoing health care’), the coefficients of the attribute *moment of payments* was not significant

A two-axes model describing the level of complexity of cost-sharing programs is used to present the key findings of the study (Fig. 1). The horizontal axis describes the attribute *moment of payments* while the vertical axis describes the attribute *type of payments*. The complete output used for the two-axes model (i.e. unweighted analyses) is shown in Tables 3, 4 and 5. As illustrated in Fig. 1, respondents preferred the same levels of each attribute, but its relative importance differed across groups (*forgoing* and *utilizing health care*). For both groups, preferences differed across the three types of health services.

**Table 3:** Results from mixed multinomial logits (prescribed medication)

Health Service:	Prescribed medication			
Group**:**	Forgoing health care		Utilizing health care	
Weighting:	Unweighted	Inverse Probability Weighting	Unweighted	Inverse Probability Weighting
	Coefficient (SE)	Coefficient (SE)	Coefficient (SE)	Coefficient (SE)
Variable**:**				
Type of payments				
-Copayments	1.224 (0.095)^**^	1.103 (0.428)^*^	1.768 (0.060)^**^	1.834 (0.237)^**^
-Coinsurances (reference)	−1.224 (0.095)^**^	−1.103 (0.428)^*^	− 1.768 (0.060)^**^	− 1.834 (0.237)^**^
Rate of payments				
-Rate1 ^a^	0.696 (0.075)^**^	0.994 (0.285)^**^	0.783 (0.033)^**^	0.901 (0.111)^**^
-Rate2 ^a^	0.257 (0.065)^**^	0.471 (0.272)	0.502 (0.027)^**^	0.500 (0.089)^**^
-Rate3 ^a^ (reference)	−0.953 (0.128)^**^	−1.466 (0.547)^**^	−1.285 (0.042)^**^	− 1.401 (0.150)^**^
Annual caps on cost-sharing payment				
-Half cap	0.015 (0.052)	0.180 (0.170)	−0.025 (0.023)	− 0.002 (0.055)
-Full cap (reference)	−0.015 (0.052)	−0.180 (0.170)	0.025 (0.023)	0.002 (0.055)
-Half cap ^*^ Copayments	−0.020 (0.049	0.030 (0.102)	−0.022 (0.017)	−0.041 (0.053)
-Half cap ^*^ Rate1 ^a^	− 0.164 (0.065)	0.072 (0.285)	− 0.053 (0.030)	−0.199 (0.088)^*^
-Half cap ^*^ Rate2 ^a^	0.040 (0.068)	−0.568 (0.408)	− 0.093 (0.036)^*^	0.070 (0.129)
-Half cap ^*^ Afterwards billed by health insurer	0.054 (0.048)	−0.119 (0.191)	0.041 (0.018)^*^	0.077 (0.070)
Moment of payment				
-Afterwards billed by health insurer	0.082 (0.045)	0.843 (0.254)^**^	1.590 (0.065)^**^	0.793 (0.198)^**^
-Directly at point of care (reference)	−0.082 (0.045)	−0.843 (0.254)^**^	−1.590 (0.065)^**^	− 0.793 (0.198)^**^
Alternative specific constant				
-Opt-out option	2.308 (0.073)^*^	2.640 (0.605)^**^	−0.332 (0.030)^**^	−1.093 (0.187)^**^
SD of random parameters				
Copayments	1.667 (0.094)^**^	1.733 (0.552)^**^	2.884 (0.080)^**^	2.650 (0.280)^**^
Rate1 ^a^	0.765 (0.108)^**^	1.036 (0.471)^*^	1.064 (0.044)^**^	1.146 (0.152)^**^
Rate2 ^a^	0.003 (0.247)	0.001 (0.005)	0.815 (0.042)^**^	−0.746 (0.149)^**^
Afterwards billed by health insurer	–	–	3.692 (0.100)^**^	3.633 (0.409)^**^
Model				
Number of individuals	475	475	6277	6277
Number of observations	8550	8550	112,986	112,986
Log-likelihood	− 2082.340	− 1327.649	−22,780.000	−16,384.221

Model: mixed multinomial logits modelling eq. 1. Random parameters assumed a normal distribution and estimated based on 5000 Halton draws

*SE* Standard Error, *SD* Standard Deviation

^a^ labels Prescribed medication: rate1 (€2/40%), rate2 (€4/50%), rate3 (€7/70%)

* = *p*-value≤.05, ** = *p*-value≤.01

**Table 4:** Results from mixed multinomial logits (diagnostic tests)

Health Service:	Diagnostic tests			
Group:	Forgoing health care		Utilizing health care	
Weighting:	Unweighted	Inverse Probability Weighting	Unweighted	Inverse Probability Weighting
	Coefficient (SE)	Coefficient (SE)	Coefficient (SE)	Coefficient (SE)
Variable**:**				
Type of payments				
-Copayments	0.664 (0.116)^**^	1.041 (0.457)^*^	0.773 (0.057)^**^	1.032 (0.234)^**^
-Coinsurances (reference)	− 0.664 (0.116)^**^	− 1.041 (0.457)^*^	− 0.773 (0.057)^**^	− 1.032 (0.234)^**^
Rate of payments				
-Rate1 ^a^	1.123 (0.102)^**^	1.758 (0.759)^*^	0.643 (0.033)^**^	0.841 (0.106)^**^
-Rate2 ^a^	0.522 (0.089)^**^	0.202 (0.350)	0.424 (0.030)^**^	0.334 (0.092)^**^
-Rate3 ^a^ (reference)	−1.646 (0.133)^**^	−1.959 (0.760)^*^	− 1.067 (0.042)^**^	−1.174 (0.150)^**^
Annual caps on cost-sharing payment				
-Half cap	−0.005 (0.055)	0.035 (0.207)	−0.003 (0.022)	0.039 (0.036)
-Full cap (reference)	0.005 (0.055)	−0.035 (0.207)	0.003 (0.022)	−0.039 (0.036)
-Half cap ^*^ Copayments	−0.050 (0.052)	−0.266 0.240)	− 0.057 (0.019)^**^	−0.084 (0.063)
-Half cap ^*^ Rate1 ^a^	0.037 (0.081)	0.227 (0.347)	−0.056 (0.033)	−0.103 (0.078)
-Half cap ^*^ Rate2 ^a^	− 0.147 (0.090)	−0.884 (0.363)^*^	− 0.019 (0.038)	− 0.053 (0.121)
-Half cap ^*^ Afterwards billed by health insurer	0.017 (0.057)	0.226 (0.231)	−0.079 (0.020)^**^	−0.221 (0.079)^**^
Moment of payment				
-Afterwards billed by health insurer	0.414 (0.086)^**^	1.581 (0.831)	3.416 (0.108)^**^	2.660 (0.390)^**^
-Directly at point of care (reference)	−0.414 (0.086)^**^	−1.581 (0.831)	−3.416 (0.108)^**^	−2.660 (0.390)^**^
Alternative specific constant				
-Opt-out option	3.752 (0.146)^**^	4.503 (1.060)^**^	0.556 (0.033)^**^	0.319 (0.247)
SD of random parameters				
Copayments	2.702 (0.143)^**^	2.599 (0.689)^**^	3.236 (0.092)^**^	3.480 (0.451)^**^
Rate1 ^a^	−1.486 (0.128)^**^	−2.132 (0.804)^**^	−0.907 (0.048)^**^	0.974 (0.161)^**^
Rate2 ^a^	1.412 (0.130)^**^	2.338 (1.035)^*^	0.827 (0.047)^**^	0.789 (0.172)^**^
Afterwards billed by health insurer	1.908 (0.113)^**^	3.829 (1.098)^**^	4.690 (0.132)^**^	4.521 (0.591)^**^
Model				
Number of individuals	738	738	5510	5510
Number of observations	13,284	13,284	99,180	99,180
Log-likelihood	− 3187.780	− 3180.645	−20,580.500	−16,919.590

Model: mixed multinomial logits modelling eq. 1. Random parameters assumed a normal distribution and estimated based on 5000 Halton draws

*SE* Standard Error, *SD* Standard Deviation

^a^ labels Diagnostic tests: rate1 (€40/15%), rate2 (€50/20%), rate3 (€80/30%)

* = *p*-value≤.05, ** = *p*-value≤.01

**Table 5:** Results from mixed multinomial logits (specialist care)

Health Service:	Specialist care			
Group:	Forgoing health care		Utilizing health care	
Weighting:	Unweighted	Inverse Probability Weighting	Unweighted	Inverse Probability Weighting
	Coefficient (SE)	Coefficient (SE)	Coefficient (SE)	Coefficient (SE)
Variable**:**				
Type of payments				
-Copayments	−0.933 (0.116)^**^	− 0.471 (0.209)^*^	− 0.622 (0.064)^**^	−0.572 (0.227)^*^
-Coinsurances (reference)	0.933 (0.116)^**^	0.471 (0.209)^*^	0.622 (0.064)^**^	0.572 (0.227)^*^
Rate of payments				
-Rate1 ^a^	0.612 (0.090)^**^	0.591 (0.180)^**^	0.508 (0.035)^**^	0.451 (0.137)^**^
-Rate2 ^a^	0.143 (0.079)	0.173 (0.129)	0.244 (0.028)^**^	0.237 (0.089)^**^
-Rate3 ^a^ (reference)	−0.755 (0.100)^**^	−0.764 (0.264)^**^	−0.752 (0.039)^**^	− 0.689 (0.143)^**^
Annual caps on cost-sharing payment				
-Half cap	0.009 (0.059)	0.010 (0.115)	−0.024 (0.022)	−0.036 (0.045)
-Full cap (reference)	−0.009 (0.059)	−0.010 (0.115)	0.024 (0.022)	0.036 (0.045)
-Half cap ^*^ Copayments	−0.075 (0.058)	−0.069 (0.117)	0.037 (0.020)	0.040 (0.063)
-Half cap ^*^ Rate1 ^a^	0.078 (0.089)	−0.023 (0.146)	−0.116 (0.034)^**^	− 0.081 (0.106)
-Half cap ^*^ Rate2 ^a^	−0.004 (0.095)	0.110 (0.175)	0.006 (0.040)	−0.032 (0.128)
-Half cap ^*^ Afterwards billed by health insurer	−0.058 (0.058)	−0.166 (0.094)	− 0.054 (0.021)^*^	0.046 (0.083)
Moment of payment				
-Afterwards billed by health insurer	0.595 (0.091)^**^	0.452 (0.135)^**^	4.086 (0.125)^**^	3.554 (0.519)^**^
-Directly at point of care (reference)	−0.595 (0.091)^**^	−0.452 (0.135)^**^	−4.086 (0.125)^**^	−3.554 (0.519)^**^
Alternative specific constant				
-Opt-out option	4.306 (0.141)^**^	1.491 (0.322)^**^	1.086 (0.036)^**^	0.545 (0.251)^*^
SD of random parameters				
Copayments	2.467 (0.123)^**^	–	3.333 (0.096)^**^	3.307 (0.445)^**^
Rate1 ^a^	−0.995 (0.133)^**^	0.855 (0.288)^**^	−0.869 (0.051)^**^	1.126 (0.168)^**^
Rate2 ^a^	0.017 (0.213)	0.001 (0.001)	−0.400 (0.075)^**^	−0.266 (0.326)
Afterwards billed by health insurer	1.801 (0.098)^**^	–	4.929 (0.139)^**^	4.478 (0.594)^**^
Model				
Number of individuals	662	662	4702	4702
Number of observations	11,916	11,916	84,636	84,636
Log-likelihood	− 2172.120	− 3287.581	−17,658.000	−12,680.018

Model: mixed multinomial logits modelling eq. 1. Random parameters assumed a normal distribution and estimated based on 5000 Halton draws

*SE* Standard Error, *SD* Standard Deviation

^a^ labels Specialist care: rate1 (€100/5%), rate2 (€140/7%), rate3 (€200/10%)

* = *p*-value≤.05, ** = *p*-value≤.01

With respect to *type of payment*, respondents preferred copayments over coinsurances for medication (coefficient (SE): 1.224 (0.095) and 1.768 (0.060)) and diagnostic tests (coefficient (SE): 0.664 (0.116) and 0.773 (0.057)), and preferred coinsurances for specialist care (coefficient (SE): 0.933 (0.116) and 0.622 (0.064)). Open-ended questions revealed that respondents were generally unaware of the actual prices of health services (a summary of answers is provided in Additional file 4). They believed that coinsurances of an unknown price would presumably result in lower fees compared to the nominal fees of copayments. With respect to *rate of payments*, respondents preferred lowest rates over the higher rates for all types of health services (coefficient (SE): for medication: 0.696 (0.075) and 0.783 (0.033); for diagnostic tests: 1.123 (0.102) and 0.643 (0.033); for specialist care: 0.612 (0.090) and 0.508 (0.035). The attribute *annual caps* was non-significant for all types of health services (coefficient (SE): for medication: 0.015 (0.052) and − 0.025 (0.023); for diagnostic tests: − 0.005 (0.055) and − 0.003 (0.022); for specialist care: 0.009 (0.059) and − 0.024 (0.022) implying that respondents’ choices were unaffected by the level of these caps. With respect to *moment of payment,* respondents generally preferred receiving a bill afterwards from the HI over direct payments at point of care (coefficient (SE): for medication: 0.082 (0.045) and 1.590 (0.065); for diagnostic tests: 0.414 (0.086) and 3.416 (0.108); for specialist care: 0.595 (0.091) and 4.086 (0.125). Respondents indicated in the open-ended questions that if payments were billed afterwards by the HI, they could arrange a payment plan with the HI and still use the health service.

The relative importance of the attributes differed between the two groups. Overall, the group *forgoing health care* considered the attribute *type of payments* as the most important whereas the group *utilizing health care* considered the attribute *moment of payments* as most important.

Results of the IPW analyses were equal to those of the unweighted analyses in terms of the direction of coefficients and preferred levels within attributes. Considering corresponding standard errors, mean coefficients in unweighted analyses were included in the range of mean and standard error of coefficients in the IPW analyses. However, note that small differences were observed for the attribute *moment of payments*, i.e. higher coefficients for the group *forgoing health care* and lower coefficients for the group *utilizing health care*.

Although not all interactions terms were significant for both groups and across all characteristics of respondents, in general, a positive correlation was observed between the utility derived from the attributes and these characteristics (i.e. utility increased when a score on the characteristic increased, and vice versa). The complete output of these models is provided in Additional file 3. For example, respondents in good health derived more utility from copayments, low rates and a bill afterwards by the HI, and thus are more likely to use health care in a given cost-sharing program than those in poor health.

### Effect of optimizing design to achieve higher adherence

If the current cost-sharing program was redesigned in accordance to respondents’ preferences of each group per type of health service, the majority of the group *forgoing health care* would choose to use health care recommended by their treating physician. Simulated rates of using health care equaled 79.8% for medication, 72.9% for diagnostic tests and 74.6% for specialist care. Higher simulated rates were found for the group *utilizing health care* (88.7% for medication; 91.3% for diagnostic tests; 92.1% for specialist care).

## Discussion

Cost-sharing programs have been introduced in many countries to increase cost-awareness among insured individuals when using health care. However, these programs have also lead to non-adherence to health care considered to be medically recommended by physicians [1] [3]. A relevant policy question is how to design these programs in such way that use of recommended care is stimulated non-differentially across subgroups. As one of the first studies, we conducted a discrete choice experiment (DCE) to assess the extent to which design characteristics of cost-sharing programs affect the decision of individuals to use care recommended by their treating physician.

The DCE revealed five main findings. First, preferences with respect to design characteristics (see Tables 3, 4 and 5) showed that less complex cost-sharing programs (i.e. copayments relative to coinsurances) improve adherence to recommended care other things being equal (ceteris paribus). Such programs offer clear information on the cost-sharing payments in advance. Furthermore, respondents preferred the afterwards billing of cost-sharing payments by the health insurer (HI) over direct payments at point of care. Although the afterwards billing by the HI causes uncertainty over the moment these payments must be paid by individuals, it does allow them to arrange a payment plan with their HI.

Second, simulations of cost-sharing programs designed in accordance with preferences of insured individuals (*optimally designed program*) revealed promising results as a large positive shift was observed for respondents who reported having forgone health care due to cost-sharing payments (group: *forgoing health care*). In the redesigned program, the majority of this group would use the recommended health service which they had previously forgone (i.e. the rate of using health care increased from 0.0% to 72.9–79.8%). For respondents who reported not having forgone health care due to cost-sharing payments (group: *utilizing health care*), a small negative shift was observed (i.e. the rate of using health care decreased from 100.0% to 88.7–92.1%) although the large majority would still use the recommended health service. Given the distribution of respondents’ characteristics across both groups (see Table 2), the redesigned program does, ceteris paribus, result in a more equal distribution of these characteristics (e.g. a lower concentration of poor health or low net income amongst individuals who forgo health care). In the context of bundled payments, policy makers may apply an *optimally designed program* to stimulate the use of in-bundle care and combine this with coinsurance fees to discourage use of out-bundle care or out-of-network providers (bundle leakage) [31].

Third, the difference in relative importance of attributes found between both groups (see Tables 3, 4 and 5) corresponds with the difference in demand-response to the current cost-sharing program (deductibles that are billed afterwards by the HI). Deductibles (similar to coinsurances) create uncertainty in the amount of expenses as they depend on actual prices. Given the most important attribute (*type of payments*) and the preference for copayments, respondents of the group *forgoing health care* were less likely to use health care in the current program (i.e. overall, coefficients had negative signs implying that coinsurances negatively affected the decision to use health care). In contrast, respondents of the group *utilizing health care* considered the attribute *moment of payments* as most important and preferred the afterwards billing of cost-sharing payments (i.e. all coefficients had positive signs). Their preferences corresponded with the current program and explain why these respondents were more likely to use health care.

Fourth, the response to cost-sharing programs may be even more heterogeneous in the real-world considering the distribution of respondents’ characteristics between the two groups (see Table 2) and the positive correlation between the utility derived from attributes and these characteristics (shown in sensitivity analyses). Estimated preferences are in line with observed real-life decisions and give credence to our study results.

Last, preferences shifted in both groups from copayments for medication and diagnostic tests, to coinsurances for specialist care (see Tables 3, 4 and 5). Analysis of open-ended questions (see Additional file 4) suggested that many respondents were unaware of actual prices of health care and were least aware of prices for specialist care. Research has reported limited price awareness in other countries where many efforts have been made to increase price transparency [32, 33]. In the Netherlands, similar initiatives have been implemented but our results suggest that price information has not reached all potential users. Our study further underscores the importance of price information as it is crucial to improve adherence to recommended care in any cost-sharing program. In the context of bundled payments, price information is also important to stimulate the use of in-bundled care (in-network providers) via cost-sharing. For example, individuals may pay an additional copayment equal to the difference in price of care provided by in-network and out-of-network providers. Individuals require price information to make well-informed decisions about the use of out-of-network providers.

### Considerations

Our study showed the extent to which design characteristics of cost-sharing programs affect the decision of individuals to use care recommended by the treating physicians. However, a few assumptions in our study may not fully hold in real-life settings. First, respondents were assumed to have basic health literacy skills, i.e. be able to obtain, process, and understand health information and services necessary to make health decisions [11]. National surveys in the US have demonstrated below-basic health literacy skills in 14% of the surveyed adults and proficient levels in 12% of this population [34]. In the Netherlands, similar levels of health literacy skills have been observed among the labor work force (11% below-basic level; 17% proficient level) [35]. An *optimally* designed cost-sharing program will improve adherence to recommended care among individuals having forgone health care due to deductibles but more interventions are required to achieve its full effect. One possible intervention is to improve patient activation; engaged individuals seek and use health information through various sources which may compensate a low-level in health literacy [11, 36].

Second, we assumed that referrals for recommended health services were solely based on the health status of individuals and did not account for possible supply-induced demand or supply-induced moral hazard. Research has shown that providers may respond to payment systems although experts consider evidence not to be completely convincing [37]. Limited research conducted in the Netherlands suggests presence of some supply-induced demand in both hospital care and GP care with differences in extent across treatments, between salaried paid physicians and those reimbursed based on output, and between subgroups of insured individuals [38, 39]. Therefore, the extent to which expected and real-life demand-response differs due to this assumption remains unclear.

Third, we assumed that use of health services will always have the same medical benefits and that not using them will always have adverse health effects. Both assumptions will unlikely be true in real-life situation.

Fourth, given our research aim, we focused solely on cost-sharing programs (i.e. design characteristics) and explicitly did not include other relevant aspects of the given health services (e.g. quality of care) as attributes. In accordance with DCE methodology, we used the ceteris paribus assumption in the choice sets by asking respondents to choose their preferred option in each choice set while keeping all other relevant factors constant. This assumption is likely to hold even if respondents implicitly correlated expenditure with quality and access, because expenditure levels differed little in our choice-sets. A systematic review focused on DCE studies concluded that in patients’ preferences with respect to cancer treatment outcome attributes (e.g. adverse effects) are considered the most important attributes relative to process (e.g. involvement in clinical decision-making) and costs (i.e. out-of-pocket costs) attributes. Hence, we expect that, if quality levels were included as attributes in our DCE, insured individuals would have been willing to pay higher cost-sharing payments for health services of high quality of care, i.e. relative importance of cost-sharing payments decreases [40].

Last, although relatively low in the Netherlands compared to those in the US, out-of-pocket payments as a share of Gross Domestic Product has increased in the last decade (from 2006 to 2015, NL 0.8–1.3%; US 2.0–1.9%). In the US, the share of adults (aged 19–64) who reported not using health care due to costs has dropped to 36% in 2014 after consecutive years of increase (from 2003 to 2012, 37–43%) [41]. Again lower than in the US, the share of Dutch citizens reporting having forgone health care due to costs is still increasing (5% in 2007; 8% in 2016) [42]. Given these trends, demand-response observed in the Dutch health system is expected to be commensurable to response observed in other health systems.

### Limitations

A few study limitations should be considered. First, our study population was possibly not representative for the Dutch population (e.g. older, higher educational levels). As shown in our IPW analyses, results may not generalizable to the whole population in terms of absolute effect magnitudes. However, the observed direction of coefficients based on a weighted representative sample remained unchanged. This indicates that our results are generalizable to the total population in terms of observed mechanism (i.e. less complex programs improve adherence to care recommended by the treating physician in all health systems). Similarly, we expect the absolute effect magnitudes to be health-system dependent (i.e. the observed magnitude of the mechanism will differ between health systems) yet we expect the underlying mechanism to be universal across health systems as the direction of demand-response to cost-sharing is considered to be universal (i.e. higher cost-sharing reduces demand of care). Moreover, although not representative for the total population, our respondents are frequent users of care and thus more likely to be faced with cost-sharing payments. This feature increases the internal validity of our findings as the observed response to cost-sharing may resemble actual behavior of individuals in real-life settings more closely.

Second, randomization was not possible in the survey tool. Instead, respondents were assigned to different blocks of the questionnaire based on regions. Although some differences in respondents’ characteristics and preferences were found across blocks, these differences did not affect our general findings.

## Conclusions

If cost-sharing programs are redesigned in such a way that individuals have clear information on cost-sharing payments in advance (i.e. copayments instead of coinsurances) and these payments are then billed by the health insurer (i.e. afterwards billed by health insurer instead of payments at point of care), adherence to recommended care may be stimulated in specific subgroups. Representative study samples are necessary to obtain absolute effect magnitudes, while this mechanism is expected to be universal across health systems. Redesigned programs provide a policy option to possibly reduce adverse health effects of cost-sharing in these groups. Considering the upcoming shift from volume-based to value-based health care provision, insights into cost-sharing program design characteristics that stimulates the use of recommended care may help to design value-based insurance plans. This study underscores the importance of price information as it is crucial to improve the use of recommended care in any cost-sharing program.

## Additional files

Additional file 1: Policy analysis. This file contains the results of the literature review and expert consultations conducted prior to this study. (DOCX 154 kb)

Additional file 2: Online questionnaire. This file contains a translated version of the online questionnaire used in this study. (DOCX 82 kb)

Additional file 3: Complete output of additional analyses. This file contains the complete output of the additional analyses (attributes are interacted with respondents’ characteristics). Each worksheet contains results from one characteristic. In each worksheet, the interactions terms are shown for easy assessment across each type of health service while the complete output is provided below. (XLSX 218 kb)

Additional file 4: Additional output. This file contains an overview of reasons for exclusion of respondents and a selection of response on open-ended questions. (DOCX 26 kb)

## Abbreviations

DCE: Discrete choice experiment
GP: General practitioner
HI: Health insurer
HIE: Health insurance experiment
MMNL: Mixed multinomial logits

## References

[1] McGuire TG. Chapter Five - Demand for Health Insurance1. In: Pauly TGM MV, Pedro PB, editors. Handbook of Health Economics, vol. 2. Amsterdam: Elsevier; 2011. p. 317–96.

[2] Cutler David M., Zeckhauser Richard J. (2000). Chapter 11 The anatomy of health insurance. Handbook of Health Economics.

[3] Newhouse JP, Group RCIE (1993). Free for all?: lessons from the RAND health insurance experiment.

[4] Fang H, Liu H, Rizzo JA (2009). Has the use of physician gatekeepers declined among HMOs? Evidence from the United States. Int J Health Care Finance Econ.

[5] Tambor M, Pavlova M, Woch P, Groot W (2010). Diversity and dynamics of patient cost-sharing for physicians’ and hospital services in the 27 European Union countries. Eur J Pub Health.

[6] Schoen C, Osborn R, Squires D, Doty MM, Pierson R, Applebaum S (2010). How health insurance design affects access to care and costs, by income, in eleven countries. Health Aff.

[7] Scott Anthony (2000). Chapter 22 Economics of general practice. Handbook of Health Economics.

[8] Briesacher BA, Gurwitz JH, Soumerai SB (2007). Patients at-risk for cost-related medication nonadherence: a review of the literature. J Gen Intern Med.

[9] Hilleman DE, Campbell JA, Bakris GL (2012). Medication adherence in heart failure. The Kidney in Heart Failure.

[10] Jacobs B, Bigdeli M, Annear PL, Van Damme W (2011). Addressing access barriers to health services: an analytical framework for selecting appropriate interventions in low-income Asian countries. Health Policy Plan.

[11] Hibbard JH, Peters E, Dixon A, Tusler M (2007). Consumer competencies and the use of comparative quality information: it isn't just about literacy. Med Care Res Rev.

[12] Sørensen K, Pelikan JM, Röthlin F, Ganahl K, Slonska Z, Doyle G, Fullam J, Kondilis B, Agrafiotis D, Uiters E (2015). Health literacy in Europe: comparative results of the European health literacy survey (HLS-EU). Eur J Pub Health.

[13] Rosenthal JA, Lu X, Cram P (2013). Availability of consumer prices from us hospitals for a common surgical procedure. JAMA Intern Med.

[14] Gerkens S, Merkur S. Belgium: health system review. In: Health systems in transition, vol. 12. Copenhagen: World Health Organization, on behalf of the European Observatory on Health Systems and Policies; 2010. p. 1–266. 21224177

[15] Kaiser Permanente. Kaiser Permanente plan: Summary of Benefits and Coverage. http://info.kaiserpermanente.org/healthplans/. Accessed 9 Oct 2018.

[16] Miller HD (2009). From volume to value: better ways to pay for health care. Health Aff.

[17] Berenson RA, Upadhyay D, Delbanco SF, Murray R (2016). Payment methods: how they work.

[18] Lancaster KJ (1966). A new approach to consumer theory. J Polit Econ.

[19] McFadden D. Conditional logit analysis of qualitative choice behavior. In: Zarembka P, editors. Frontiers in Econometrics. New York City: Academic Press; 1974. p. 105–142.

[20] Louviere JJ, Hensher DA, Swait JD (2000). Stated choice methods: analysis and applications.

[21] Pearlin Leonard I., Menaghan Elizabeth G., Lieberman Morton A., Mullan Joseph T. (1981). The Stress Process. Journal of Health and Social Behavior.

[22] Pudrovska T, Schieman S, Pearlin LI, Nguyen K (2005). The sense of mastery as a mediator and moderator in the association between economic hardship and health in late life. J Aging Health.

[23] Orme B (1998). Sample size issues for conjoint analysis studies. Sawthooth Software Research paper Series.

[24] Johnson R, Orme B (2003). Getting the most from CBC. Sawtooth Softw Res Paper Ser..

[25] Bech M, Gyrd-Hansen D (2005). Effects coding in discrete choice experiments. Health Econ.

[26] Cole SR, Hernán MA (2008). Constructing inverse probability weights for marginal structural models. Am J Epidemiol.

[27] Statistics Netherlands (CBS). StatLine. https://opendata.cbs.nl/statline/#/CBS/nl/. Accessed 3 July 2018.

[28] Kolenikov S (2014). Calibrating survey data using iterative proportional fitting (raking). Stata J.

[29] SAS Institute Inc. SAS/STAT®9.3. Cary: SAS Institute Inc.; 2012.

[30] StataCorp (2015). Stata Statistical Software: Release 14.

[31] Gunderman RB, Patti JA, Lexa F, Weinreb J, Hillman BJ, Thrall JH, Neiman H (2010). The 2009 ACR forum: health care payment models. J Am Coll Radiol.

[32] NHS. Reference Costs 2015-16;. London: Department of Health and Social Care; 2016. https://www.gov.uk/government/publications/nhs-reference-costs-2015-to-2016. Accessed 9 Oct 2018.

[33] Public Agenda. How people use health care Price information in the United States, New York State, Florida, Texas and New Hampshire. New York City: Public Agenda; 2017. https://www.publicagenda.org/files/PublicAgenda_StillSearching_2017.pdf. Accessed 9 Oct 2018.

[34] Kutner M, Greenburg E, Jin Y, Paulsen C. The Health Literacy of America's Adults: Results from the 2003 National Assessment of Adult Literacy. NCES 2006–483. Washington, DC: National Center for Education Statistics; 2006.

[35] Fransen M, Stronks K, Essink-Bot M, Geneeskunde AS, Essink M. Gezondheidsvaardigheden: Stand van zaken. In: AMC, Universiteit van Amsterdam, Afd Sociale Geneeskunde, vol. 17. The Hague: Gezondheidsraad, Laaggeletterdheid te lijf, Publicatienummer; 2011.

[36] Hibbard JH, Stockard J, Mahoney ER, Tusler M (2004). Development of the patient activation measure (PAM): conceptualizing and measuring activation in patients and consumers. Health Serv Res.

[37] Chandra Amitabh, Cutler David, Song Zirui (2011). Who Ordered That? The Economics of Treatment Choices in Medical Care. Handbook of Health Economics.

[38] Douven RCMH, Mocking R, Mosca I. The effect of physician fees and density differences on regional variation in hospital treatments. The Hague: CPB Netherlands Bureau for Economic Policy Analysis; 2012.

[39] Dijk CE, Berg B, Verheij RA, Spreeuwenberg P, Groenewegen PP, Bakker DH (2013). Moral Hazard and supplier-induced demand: empirical evidence in general practice. Health Econ.

[40] Bien DR, Danner M, Vennedey V, Civello D, Evers SM, Hiligsmann M (2017). Patients’ preferences for outcome, process and cost attributes in Cancer treatment: a systematic review of discrete choice experiments. Patient Centered Outcomes Res.

[41] Collins SR, Rasmussen PW, Doty MM, Beutel S (2015). The rise in health care coverage and affordability since health reform took effect: findings from the Commonwealth Fund Biennial Health Insurance Survey, 2014. Issue Brief (Commonw. Fund).

[42] Van der Wees PJ, Wammes JJG, Westert GP. International Health Policy Survey 2016. Nijmegen: IQ healthcare; 2016. http://www.iqhealthcare.nl/media/124472/20170607_def_rapportage_cmwf_survey_2016.pdf. Accessed 9 Oct 2018.

