# Supplementary material for: The effect of cost-sharing design characteristics on use of health care recommended by the treating physician; a discrete choice experiment
Source: BMC Health Serv Res. 2018 Oct 20;18:797. doi: 10.1186/s12913-018-3598-4 (PMC6195970; doi:10.1186/s12913-018-3598-4)
Supplement: Supplementary file 1 — Policy analysis. This file contains the results of the literature review and expert consultations conducted prior to this study. (DOCX 154 kb) [file 12913_2018_3598_MOESM1_ESM.docx]

# Additional file 1, Policy analysis

To understand which characteristics of a cost-sharing program reduce its complexity, we have examined cost-sharing programs implemented in a number of developed countries from the perspective of an insured individual. This involved a review of the literature provided and consultations of experts working at different stakeholders in healthcare, i.e. health insurance companies, patient organization, health policy makers, consultants specialized in financial transactions and researchers.

In the first step, common daily-life financial transactions (e.g. buying grocery) were compared to those in healthcare from an insured individual perspective [1]. A key difference when making a transaction is that in the former situation there is no uncertainty in how much the consumer has to pay and at what moment, while in healthcare the actual amount and the moment of payment remains uncertain for the average insured individual. In the second step, we consulted patient organizations concerning our analysis who confirmed that these two elements (*amount of cost-sharing payments* and *moment of payment*) induce uncertainty and may affect the decision to adhere to recommended care by physicians. In the final step, we examined cost-sharing programs implemented in a number of developed countries and identified four design characteristics that affect complexity of cost-sharing payments, 1) *the* *type of cost-sharing payments*, 2) *the* *rate of cost-sharing payments*, 3) *annual caps on cost-sharing*, and 4) *the* *moment that cost-sharing payments must be paid*. Our research showed that most cost-sharing programs in developed countries involve different combinations of these four characteristics.

To describe the level of complexity observed in existing cost-sharing programs, we plotted a two-axes models (figure 2 and 3). The horizontal axis describes the design characteristic (directly paid at point of care or billed afterwards by the health insurer (HI)) while the vertical axis describes the design characteristic *type of payments* (payments consisting of copayments or coinsurances). The two other design characteristics are not plotted in the model. With respect to the design characteristic *rate of cost-sharing* *payments*, the effects of uncertainty are equal across different rate levels according of the panel of consulted experts. For example, a low flat fee of €5 holds the same level of uncertainty in payments as a higher flat fee of €40. This also holds for an proportional fee of 5% compared to a proportional fee of 10%. The proportional fees hold more uncertainty compared to flat fees but is captured by the design characteristic *type of cost-sharing payments*. With respect to *annual caps* *on cost-sharing*, once patients have paid cost-sharing equal to these caps any financial barrier to use healthcare is lifted regardless of its design. Although the ceiling of a cap may vary, it does not increase or reduce uncertainty according to the panel of consulted experts. In some countries (e.g. Israel), cost-sharing payments made by individuals are directly recorded in a national database which provides individuals, health insurers and health providers immediate full insight of all caps applicable to the individuals’ situation and remaining level of these caps [2]. Hence, upgrading the IT infra-structure of billing systems is more likely to reduce complexity resulting from annual caps on cost-sharing. Table 4 provides a summary of annual caps in various programs.


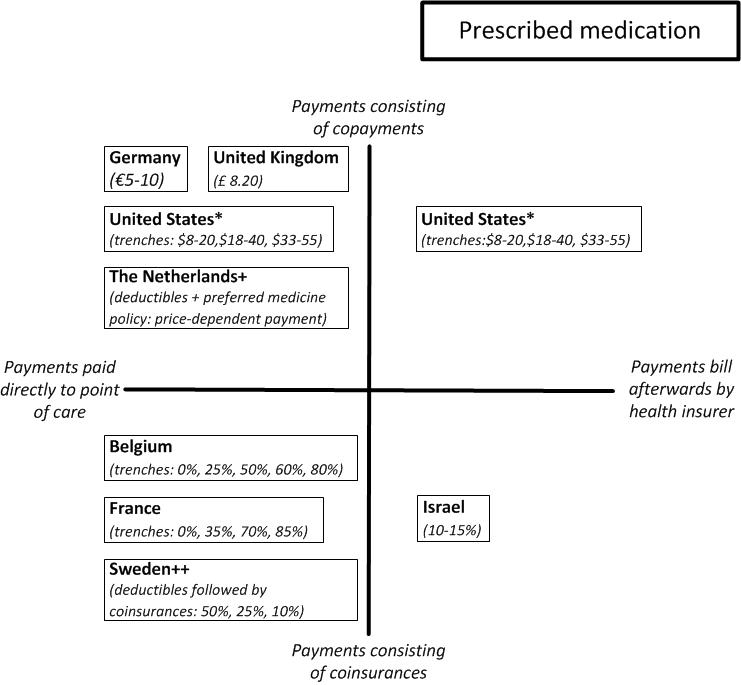


Figure 2, Cost-sharing programs for prescribed medication in multiple countries.

**depends on health reimbursement arrangement (e.g. automatic reimbursement or health payment card), +deductibles are combined with preferred medicine policy (e.g. individuals pay the reference price of a drug as deductible payments and an additional copayment equal to the difference in the price of the drug and reference drug, ++deductibles followed by coinsurances which gradually decreases (e.g. once the ceiling of the deductible (€122) is reached, individuals pay 50% of the costs between €122-233), 25% of the costs between €233-433 and 10% of the costs between €433-600). Figure is based on authors’ analysis of a literature review [2-9] and the panel of consulted experts.*


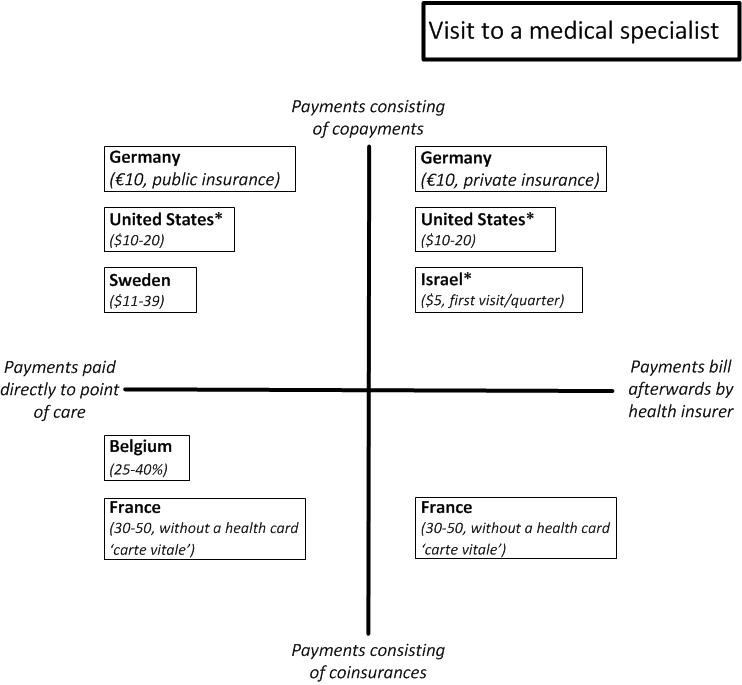


Figure 3, Cost-sharing programs for a visit to a medical specialist in multiple countries.

**depends on health reimbursement arrangement (e.g. automatic reimbursement or health payment card). Figure is based on authors’ analysis of a literature review [2-9] and the panel of consulted experts.*

Table 6, Summary of cost-sharing caps in various countries.

| Belgium | An household income dependent preferential reimbursement policy is applicable. In 2009, household with an gross annual income below €14,339.94 + €2,654.70 per dependent receive lower coinsurance rates. In addition, different maximum limits (MAB) apply after which copayments are reimbursed; €450 for vulnerable groups (e.g. low-income, disabled individuals), €650 (children<19 years) and income dependent limit €450-1800. |
| --- | --- |
| Germany | Different funds offer lower deductibles and no-claim bonuses for specific groups. Children younger than 18 years are exempted.  In addition, cost-sharing is limited to 2% of the household income (1% for individuals qualifying as chronically ill) |
| France | Cost-sharing may be reimbursed with a complementary private health insurance.  An annual ceiling of €50 applies for all cost-sharing.  Individuals with one of the 32 chronic illnesses are exempted from cost-sharing of treatment required for their conditions.  Children and low-income individuals are exempted from cost-sharing. |
| UK | Children younger than 16 years old, low-income individuals, individuals older than 60 years, pregnant women and those who have had a baby in the 12 months, individuals with cancer and specific long-term conditions and disabilities are exempted from cost-sharing.  Individual may purchase an additional insurance to limit cost-sharing of medication to £2 per week. |
| US | Annual out-of-pocket costs is limited to $3,500 per person or $9,400 per family. Cost-sharing is exempted for specific health services for specific groups (e.g. prenatal and postnatal care for pregnant women). |
| The Netherlands | An annual deductible of €385 applies for most health services covered by the basic health package. Children younger than 18 years are exempted as well as few health services (e.g. General Practitioners care, maternity care). |
| Sweden | Most county councils exempt individuals under the age of 20 years from cost-sharing. The annual cap for healthcare visits is € 122. Annual caps are separated for various health services (e.g. prescribed medication €244, medical devices €220). |
| Israel | Elderly welfare recipients (65 years and older), children receiving disability payments are exempt from copayments for all visits. Individuals with certain medical conditions (e.g. cancer, HIV/AIDS) are exempt from copayments at outpatient hospital care. Cost-sharing is also capped at household level per quarter (e.g. €50 per household, €75 for individuals with chronic conditions, 50% lower for elderly people). |

Table is based on authors’ analysis of a literature review [2-9] and the panel of consulted experts.

As illustrated in figure 2 and 3, cost-sharing programs of prescribed medication and visits to a medical specialist are located across all four quadrants of the models. Programs located in the upper left quadrant hold relatively the lowest level of uncertainty according to the panel of consulted experts and reviewed literature (fixed flat fees that are paid directly at point of care). In contrast, programs located in the bottom right quadrant hold the highest level of uncertainty (coinsurances dependent on unknown prices resulting in an unknown amount of expenses that are billed afterwards at an unknown moment in time). Our policy analysis confirmed that cost-sharing programs vary across countries and may be classified using the four design characteristics (*the* *type of cost-sharing payments*, *the* *rate of cost-sharing payments*, *annual caps on cost-sharing*, and *the* *moment that cost-sharing payments must be paid*).

**References**

1. van der Hijden E, Lycklama D: Schud de consument in de patiënt wakker. In: De Trouw. 2015.

2. Rosen B, Waitzberg R, Merkur S: Israel: Health System Review. Health Syst Transit 2015, 17(6):1-212.

3. Busse R, Blümel M: Health systems in transition. Health 2014, 16(2).

4. Cylus J, Richardson E, Findley L, Longley M, O'Neill C, Steel D: United Kingdom: Health System Review. Health Syst Transit 2015, 17(5):1-126.

5. Rice T, Rosenau P, Unruh LY, Barnes AJ, Saltman RB, van Ginneken E: United States of America: health system review. Health Syst Transit 2013, 15(3):1-431.

6. Kroneman M, Boerma W, van den Berg M, Groenewegen P, de Jong J, van Ginneken E: Netherlands: Health System Review. Health systems in transition 2016, 18(2):1-240.

7. Gerkens S, Merkur S: Belgium: Health system review. In: Health systems in transition. vol. 12; 2010: 1-266.

8. Anell A, Glenngard AH, Merkur S: Sweden health system review. Health Syst Transit 2012, 14(5):1-159.

9. Chevreul K, Berg Brigham K, Durand-Zaleski I, Hernandez-Quevedo C: France: Health System Review. Health Syst Transit 2015, 17(3):1-218, xvii.
