# Supplementary material for: The effect of cost-sharing design characteristics on use of health care recommended by the treating physician; a discrete choice experiment
Source: BMC Health Serv Res. 2018 Oct 20;18:797. doi: 10.1186/s12913-018-3598-4 (PMC6195970; doi:10.1186/s12913-018-3598-4)
Supplement: Supplementary file 2 — Online questionnaire. This file contains a translated version of the online questionnaire used in this study. (DOCX 82 kb) [file 12913_2018_3598_MOESM2_ESM.docx]

All panel members of the Dutch Patient Federation were invited by email to complete the questionnaire. In this email, we informed panel members that participation was voluntary and could be terminated at any time during the questionnaire. In addition, their contributions would be made anonymous, would not be traced back to individuals and would be used for academic publication. We indicated that respondents gave informed consent by accessing the online questionnaire via the URL included in the email. Based on these conditions, approval by the ‘Dutch Medical Research Involving Human Subjects Act’ was not applicable.

We generated different versions of the questionnaire (version 1-4) to accommodate the four blocks of our experimental design. In interest of brevity, we included a single version (version 1 only) as the questions are equal across all versions with the exception of the presented choice sets due to the different blocks.

After the introduction page, respondents were assigned to one of the four versions based on regions. After the introductory questions, respondents were divided into two groups (question 5). As described in the methods section, respondents were instructed to use this situation as context situation in the DCE, i.e. (a) they had forgone health care recommended by the treating physician due to deductibles payments within the last two years, or (b) they had used health care recommended by the treating physician and subject to deductibles payments. To accommodate the difference in context situations, two subversions of the questionnaire (subversion A and B) were generated. Respondents in the group *forgoing health care* were presented subversion A, while respondents in the group *utilizing health care* received subversion B. The questionnaire ended with questions regarding demographics and feedback.

# Introduction page

| Newspapers have recently reported that some patients do not adhere to medical treatment recommended by their treating physician due to payments of the mandatory deductible.  We define recommended care as medical care that a physician considers necessary given your health status. Examples are prescribed medicines, ordered blood tests, ordered x-rays, referred visits to or treatment by a medical specialist in a hospital.  The mandatory deductible is a fixed amount that all Dutch adults have to pay if they have used healthcare covered by the basic health insurance package. Several health services (e.g. visits to a General Practitioner) are not subject to the deductible set at €385 in 2016.  **Goal**  This questionnaire assesses the effects of mandatory deductible payments on patients’ use of health care recommended by their treating physician.  We want to know what types of health care and how often patients forgo them due to deductible payments. Additionally, we would also like to know what patients will do if a new cost-sharing payment scheme is introduced. Does it affect use of health care?  **Questionnaire**  We start with a several questions concerning the mandatory deductible followed by the question whether you have forgone recommended care due to deductible payments in the last two years. Hereafter, you are asked to state what you do if new cost-sharing payment schemes are introduced. The questionnaire ends with general questions about age, gender and health.  Expected duration to complete: 20 minutes  Thank you for your time. |
| --- |

# Region question

| 1. In which province do you live? 2. Drenthe 3. Flevoland 4. Friesland 5. Gelderland 6. Groningen 7. Limburg 8. Noord-Brabant 9. Noord-Holland 10. Overijssel 11. Utrecht 12. Zeeland 13. Zuid-Holland |
| --- |

# Introductory questions vocational

| 1. How much of your mandatory deductible did you spend in 2015? 2. I spent all of my mandatory deductible. 3. I spent a part of my mandatory deductible. 4. I did not use my mandatory deductible. 5. I do not know. |
| --- |
| 1. How much of your mandatory deductible do you expect to spend in 2016? 2. I expect to have fully spent my mandatory deductible. 3. I expect to have spent part of my mandatory deductible. 4. I do not expect to have used my mandatory deductible. 5. I do not know yet. |
| Many health insurers provide arrangements for people who are unable to pay their mandatory deductible as a single payment. These people pay a monthly fixed amount (1/12^th^ of €385). If they use health care subject to the mandatory deductible during that period, the required payments are deducted from the ‘saved’ amount they receive any remainders back.   1. Do you currently pay the mandatory deductible in monthly terms to your health insurer? 2. Yes. 3. No, but I would like to. 4. No, I do not need that. 5. Otherwise, namely…… [text box] |

# Group classification (version A or B)

| 1. Have you forgone any health care recommended by a physician in the past two years due to mandatory deductible payments?   *Note: visits to your General Practitioner do not apply as these visits are not subject to mandatory deductible payments.*   1. Yes, I have forgone health care due to mandatory deductible payments. 2. No, I have not forgone health care due to mandatory deductible payments. 3. I do not know whether I have forgone health care due to mandatory deductible payments. |
| --- |

# Version 1A1 Prescribed Medication – Group: *Forgoing health care*

| 1. Have you forgone prescribed medicines due to mandatory deductible payments in the past two years? 2. Yes, I have forgone prescribed medicines due to mandatory deductible payments. 3. No. |
| --- |
| 1. How often did this occur on average? 2. Less than 1 time per year. 3. 1-2 times per year. 4. 3-5 times per year. 5. 6-12 times per year. 6. More than 12 times per year. |
| 1. Why were the mandatory deductible payments a reason to forgo prescribed medicines? *Multiple answers possible.* 2. I could not afford it. 3. I was not willing to pay for it. 4. Otherwise, namely…… [text box] |
| Use the most recent occurrence in which you had forgone prescribed medicines due to mandatory deductible payments when answering the next questions.   1. Which type of prescribed medicines have you forgone? 2. Pain relievers. 3. Cholesterol-lowering medication. 4. Stomach acid inhibitors. 5. Anti-hypertensives. 6. Antibiotics / Penicillin. 7. Diuretics. 8. Asthma medication / COPD medication. 9. Eczema medication. 10. Blood thinners. 11. Diabetes medication. 12. Anti-epileptic drugs. 13. Antidepressants. 14. Hay fever medication. 15. Otherwise, namely…… [text box] 16. I do not know anymore. |
| 1. Did you buy an alternative remedy somewhere else? *For example, an alternative without prescription at a local drugstore.* 2. Yes. 3. No. 4. I do not know anymore. |
| 1. Do you know what you would have to pay due to mandatory deductibles if you had picked up the prescribed medicines? *Enter a rounded amount in euros.* 2. Yes, namely…… [text box] 3. No, I do not know that. |
| 1. We would like to know about any other reasons why you had forgone prescribed medicines.   [text box] |

| **The current payment system**  If you use health care covered by the mandatory basic health insurance package and subject to the mandatory deductible, you have to pay deductible payments up to 385 euros in 2016.  Prescribed medicines are covered by the basic health insurance package and you have to pay (part) of their costs depending on any previous payments due to the mandatory deductible. When you fill your prescription at the pharmacy, you generally do not pay the pharmacy directly. Your health insurer pays the pharmacy and sends you a bill for cost-sharing payments afterwards.  The health insurer sends the bill to you afterwards. This may take several weeks to months. If you pick up medicines at the pharmacy, you often do not know much you have to pay as this depends on, for example, on the quantity and price of the medicines.  **New payment system**  Suppose that the government considers a new system that replaces the current system of mandatory deductibles. In this new system, annual cost-sharing payments are capped at €385 and two different types of payments are possible:   - A fixed amount per box of medicines (e.g. €4 per box). - A percentage of the actual price per box of medicines (e.g. 50% of the price).   In this new system you pay either directly to the pharmacy or you are billed afterwards by your health insurer for this payment. You have the option to forgo health care by choosing not to fill the medicines (e.g. if you cannot or do not want to pay for the medicines).  **Following questions**  In the following questions, a new system is described and three options are provided. We would like you to indicate which of these options you prefer the most if the current system of mandatory deductibles is replaced.  Use the most recent occurrence in which you had forgone prescribed medicines due to mandatory deductible payments when answering these questions. |
| --- |
| **An example question**  Consider your most recent situation in which you did not pick up medicines. Assume that a new system has been introduced which applies an annual cap set at €385 and that you have not used health care subject to cost-sharing yet.  Which option do you prefer?   1. Directly pay 2 euros per medicine box to the pharmacy. 2. Pay 70% of the price per medicine box to the health insurer (billed afterwards). 3. Choose not to pick up the medicines (forgo health care, no cost-sharing payments).   *Explanation*  Option (A) implies that you pay 2 euros per box of medicines and have to pay this directly to the pharmacist. Option (B) implies that you pay 70% of the price per medicine box, but you are billed for this payment by the health insurer afterwards (i.e. you pick up the medicines and receive the bill later at an unknown moment). Option (C) implies that you choose not to pick up the medicines and you do not have to pay. |

| Use the most recent occurrence in which you had forgone prescribed medicines due to mandatory deductible payments when answering the next questions. Please indicate which of the possible options you prefer in each situation: |
| --- |
| 1. Situation 1*   Consider your most recent situation in which you did not pick up medicines. Assume that a new system has been introduced which applies an annual cap set at €385 and that you have not used health care subject to cost-sharing yet.  Which option do you prefer?   1. Directly pay 50% of the price per medicine box to the pharmacy. 2. Pay 7 euros per medicine box to the health insurer (billed afterwards). 3. Choose not to pick up the medicines (forgo health care, no cost-sharing payments). |
| 1. Situation 2*   Consider your most recent situation in which you did not pick up medicines. Assume that a new system has been introduced which applies an annual cap set at €385 and that you have not used health care subject to cost-sharing yet.  Which option do you prefer?   1. Pay 70% of the price per medicine box the health insurer (billed afterwards). 2. Directly pay 2 euros per medicine box to the pharmacy. 3. Choose not to pick up the medicines (forgo health care, no cost-sharing payments). |
| 1. Situation 3*   Consider your most recent situation in which you did not pick up medicines. Assume that a new system has been introduced which applies an annual cap set at €385 and that you have not used health care subject to cost-sharing yet.  Which option do you prefer?   1. Directly pay 2 euros per medicine box to the pharmacy. 2. Pay 50% of the price per medicine box to the health insurer (billed afterwards). 3. Choose not to pick up the medicines (forgo health care, no cost-sharing payments). |

| Use the most recent occurrence in which you had forgone prescribed medicines due to mandatory deductible payments when answering the next questions. Please indicate which of the possible options you prefer in each situation: |
| --- |
| 1. Situation 4*   Consider your most recent situation in which you did not pick up medicines. Assume that a new system has been introduced which applies an annual cap set at €385 and that you have paid cost-sharing payments equal to half of the annual cap.  Which option do you prefer?   1. Pay 2 euros per medicine box to the health insurer (billed afterwards). 2. Directly pay 70% of the price per medicine box to the pharmacy. 3. Choose not to pick up the medicines (forgo health care, no cost-sharing payments). |
| 1. Situation 5*   Consider your most recent situation in which you did not pick up medicines. Assume that a new system has been introduced which applies an annual cap set at €385 and that you have paid cost-sharing payments equal to half of the annual cap.  Which option do you prefer?   1. Directly pay 4 euros per medicine box to the pharmacy. 2. Pay 40% of the price per medicine box to the health insurer (billed afterwards). 3. Choose not to pick up the medicines (forgo health care, no cost-sharing payments). |
| 1. Situation 6*   Consider your most recent situation in which you did not pick up medicines. Assume that a new system has been introduced which applies an annual cap set at €385 and that you have paid cost-sharing payments equal to half of the annual cap.  Which option do you prefer?   1. Directly pay 70% of the price per medicine box to the pharmacy. 2. Pay 4 euros per medicine box to the health insurer (billed afterwards). 3. Choose not to pick up the medicines (forgo health care, no cost-sharing payments). |

| Different fixed amounts and percentages were used in the previous questions. Fixed amounts consisted of 2, 4 and 7 euros per medicine box. Proportional amounts reflected 40%, 50% or 70% of the price per medicine box.  It has been reported that, on average, patients do not know the costs of their medications. We would like to know more about this.  Use the most recent occurrence in which you had forgone prescribed medicines due to mandatory deductible payments when answering the next questions. |
| --- |
| 1. Do you know the average costs of your medicines?   *If so, enter a rounded amount in euros……* [text box] |
| 1. Do you think that the different fixed amounts and percentages used in the previous questions are: 2. Way too high. 3. Too high. 4. Just right. 5. Too low. 6. Way too low. |
| 1. We would like to know any additional explanations for your previous answers. We would also like to know any other reasons why you had forgone medicines. If desired, you may also make any other comments regarding forgoing health care.   [text box] |

# Version 1A2 Diagnostic tests – Group: *Forgoing health care*

| 1. Have you forgone diagnostic tests due to mandatory deductible payments in the past two years? 2. Yes, I have forgone diagnostic tests due to mandatory deductible payments. 3. No. |
| --- |
| 1. How often did this occur on average? 2. Less than 1 time per year. 3. 1-2 times per year. 4. 3-5 times per year. 5. 6-12 times per year. 6. More than 12 times per year. |
| 1. Why were the mandatory deductible payments a reason to forgo diagnostic tests? *Multiple answers possible.* 2. I could not afford it. 3. I was not willing to pay for it. 4. Otherwise, namely…… [text box] |
| Use the most recent occurrence in which you had forgone diagnostic tests due to mandatory deductible payments when answering the next questions.   1. Which type of diagnostic tests have you forgone? 2. Blood tests. 3. Urinary or faeces tests. 4. X-rays, a CAT or MRI scan, ultrasound test. 5. Cycling test or lung function test. 6. Otherwise, namely…… [text box] 7. I do not know anymore. |
| 1. Do you know what you would have to pay due to mandatory deductibles if you had undergone the diagnostic tests? *Enter a rounded amount in euros.* 2. Yes, namely…… [text box] 3. No, I do not know that. |
| 1. Do you think that the different fixed amounts and percentages used in the previous are: 2. Way too high. 3. Too high. 4. Just right. 5. Too low. 6. Way too low. |
| 1. We would like to know any additional explanations for your previous answer. We would also like to know any other reasons why you had forgone diagnostic tests.   [text box] |

| **The current payment system**  If you use health care covered by the mandatory basic health insurance package and subject to the mandatory deductible, you have to pay deductible payments up to 385 euros in 2016.  Diagnostic tests are covered by the mandatory basic health insurance package and you have to pay (part) of their costs depending on any previous payments due to the mandatory deductible. When you undergo diagnostic tests at a hospital, you generally do not pay the hospital directly. Your health insurer pays the hospital and sends you a bill for cost-sharing payments afterwards.  The health insurer sends the bill to you afterwards. This may take several weeks to months. If you undergo diagnostic tests at a hospital, you often do not know much you have to pay as this depends on, for example, on the type and price of the examination.  **New payment system**  Suppose that the government considers a new system that replaces the current system of mandatory deductibles. In this new system, annual cost-sharing payments are capped at €385 and two different types of payments are possible:   - A fixed amount per examination (e.g. €50 per blood test examination or per CAT scan). - A percentage of the actual price per examination (e.g. 30% of the price).   In this new system you pay either directly to the hospital or you are billed afterwards by your health insurer for this payment. You have the option to forgo health care by choosing not to undergo the diagnostic tests (e.g. if you cannot or do not want to pay for the examination).  **Following questions**  In the following questions, a new system is described and three options are provided. We would like you to indicate which of these options you prefer the most if the current system of mandatory deductibles is replaced.  Use the most recent occurrence in which you had forgone diagnostic tests due to mandatory deductible payments when answering the next questions. |
| --- |
| **An example question**  Consider your most recent situation in which you did not undergo diagnostic tests. Assume that a new system has been introduced which applies an annual cap set at €385 and that you have not used health care subject to cost-sharing yet.  Which option do you prefer?   1. Directly pay 40 euros per examination to the hospital. 2. Pay 30% of the examination’s price to the health insurer (billed afterwards). 3. Choose not to undergo the examination (forgo health care, no cost-sharing payments).   *Explanation*  Option (A) implies that you pay 40 euros per examination and have to pay this directly to the hospital. Option (B) implies that you pay 30% of the price per examination, but you are billed for this payment by the health insurer afterwards (i.e. you undergo the examination and receive the bill later at an unknown moment). Option (C) implies that you choose not to undergo the examination and you do not have to pay. |

| Use the most recent occurrence in which you had forgone diagnostic tests due to mandatory deductible payments when answering the next questions. Please indicate which of the possible options you prefer in each situation: |
| --- |
| 1. Situation 1*   Consider your most recent situation in which you did not undergo diagnostic tests. Assume that a new system has been introduced which applies an annual cap set at €385 and that you have not used health care subject to cost-sharing yet.  Which option do you prefer?   1. Pay 20% of the examination’s price to the health insurer (billed afterwards). 2. Directly pay 80 euros per examination to the hospital. 3. Choose not to undergo the examination (forgo health care, no cost-sharing payments). |
| 1. Situation 2*   Consider your most recent situation in which you did not undergo diagnostic tests. Assume that a new system has been introduced which applies an annual cap set at €385 and that you have not used health care subject to cost-sharing yet.  Which option do you prefer?   1. Directly pay 30% of the examination’s price to the hospital. 2. Pay 40 euros per examination to the health insurer (billed afterwards). 3. Choose not to undergo the examination (forgo health care, no cost-sharing payments). |
| 1. Situation 3*   Consider your most recent situation in which you did not undergo diagnostic tests. Assume that a new system has been introduced which applies an annual cap set at €385 and that you have not used health care subject to cost-sharing yet.  Which option do you prefer?   1. Directly pay 15% of the examination’s price to the hospital. 2. Pay 50 euros per examination to the health insurer (billed afterwards). 3. Choose not to undergo the examination (forgo health care, no cost-sharing payments). |

| Use the most recent occurrence that you had forgone diagnostic tests due to mandatory deductible payments when answering the next questions. Please indicate which of the possible options you prefer in each situation: |
| --- |
| 1. Situation 4*   Consider your most recent situation in which you did not undergo diagnostic tests. Assume that a new system has been introduced which applies an annual cap set at €385 and that you have paid cost-sharing payments equal to half of the annual cap.  Which option do you prefer?   1. Pay 15% of the examination’s price to the health insurer (billed afterwards). 2. Directly pay 50 euros per examination to the hospital. 3. Choose not to undergo the examination (forgo health care, no cost-sharing payments). |
| 1. Situation 5*   Consider your most recent situation in which you did not undergo diagnostic tests. Assume that a new system has been introduced which applies an annual cap set at €385 and that you have paid cost-sharing payments equal to half of the annual cap.  Which option do you prefer?   1. Pay 80 euros per examination to the health insurer (billed afterwards). 2. Directly pay 20% of the examination’s price to the hospital. 3. Choose not to undergo the examination (forgo health care, no cost-sharing payments). |
| 1. Situation 6*   Consider your most recent situation in which you did not undergo diagnostic tests. Assume that a new system has been introduced which applies an annual cap set at €385 and that you have paid cost-sharing payments equal to half of the annual cap.  Which option do you prefer?   1. Directly pay 15% of the examination’s price to the hospital. 2. Pay 80 euros per examination to the health insurer (billed afterwards). 3. Choose not to undergo the examination (forgo health care, no cost-sharing payments). |

| Different fixed amounts and percentages were used in the previous questions. Fixed amounts consisted of 40, 50 and 80 euros per examination. Proportional amounts reflected 15%, 20% or 30% of the examination’s price.  It has been reported that, on average, patients do not know the costs of their diagnostic tests. We would like to know more about this.  Use the most recent occurrence in which you had forgone diagnostic tests due to mandatory deductible payments when answering the next questions. |
| --- |
| 1. Do you know the average costs of your diagnostic tests?   *If so, enter a rounded amount in euros……* [text box] |
| 1. Do you think that the different fixed amounts and percentages used in the previous questions are: 2. Way too high. 3. Too high. 4. Just right. 5. Too low. 6. Way too low. |
| 1. We would like to know any additional explanations for your previous answers. We would also like to know any other reasons why you had forgone diagnostic tests. If desired, you may also make any other comments regarding forgoing health care.   [text box] |

# Version 1A3 Specialist care – Group: *Forgoing health care*

| 1. Have you forgone visits to or treatment by the medical specialists in the hospital (i.e. specialist care) due to mandatory deductible payments in the past two years? 2. Yes, I have forgone prescribed medicines due to mandatory deductible payments. 3. No. |
| --- |
| 1. How often did this occur on average? 2. Less than 1 time per year. 3. 1-2 times per year. 4. 3-5 times per year. 5. 6-12 times per year. 6. More than 12 times per year. |
| 1. Why were the mandatory deductible payments a reason to forgo specialist care? *Multiple answers possible.* 2. I could not afford it. 3. I was not willing to pay for it. 4. Otherwise, namely…… [text box] |
| Use the most recent occurrence in which you had forgone specialist care due to mandatory deductible payments when answering the next questions.   1. For which specialism have your forgone specialist care? *[dropdown menu]* 2. Cardiology. 3. Dental surgery. 4. Dermatology. 5. ENT medicine. 6. Ophthalmology. 7. Emergency Room. 8. Gastrointestinal medicine. 9. General surgery. 10. Gynaecology. 11. Haematology. 12. Internal medicine. 13. Nephrology. 14. Neurology. 15. Orthopaedics. 16. Pulmonology. 17. Radiotherapy. 18. Rehabilitation medicine. 19. Rheumatology. 20. Otherwise, namely…… [text box] 21. No, I do not know that. |
| 1. Do you know what you would have to pay due to mandatory deductibles if you had used the specialist care? *Enter a rounded amount in euros.* 2. Yes, namely…… [text box] 3. No, I do not know that. |
| 1. We would like to know reasons why you had forgone specialist care.   [text box] |

| **The current payment system**  If you use health care covered by the mandatory basic health insurance package and subject to the mandatory deductible, you have to pay deductible payments up to 385 euros in 2016.  Specialist care is covered by the mandatory basic health insurance package and you have to pay (part) of its costs depending on any previous payments due to the mandatory deductible. When you use specialist care at a hospital, you generally do not pay the hospital directly. Your health insurer pays the hospital and sends you a bill for cost-sharing payments afterwards.  The health insurer sends the bill to you afterwards. This may take several weeks to months. If you use specialist care at a hospital, you often do not know much you have to pay as this depends on, for example, on the diagnosis, the type and price of the specialist care.  **New payment system**  Suppose that the government considers a new system that replaces the current system of mandatory deductibles. In this new system, annual cost-sharing payments are capped at €385 and two different types of payments are possible:   - A fixed amount per visit or treatment (e.g. €140 per visit or treatment). - A percentage of the actual price per visit or treatment (e.g. 7% of the price).   In this new system you pay either directly to the hospital or you are billed afterwards by your health insurer for this payment. You have the option to forgo health care by choosing not to use specialist care (e.g. if you cannot or do not want to pay for the specialist care).  **Following questions**  In the following questions, a new system is described and three options are provided. We would like you to indicate which of these options you prefer the most if the current system of mandatory deductibles is replaced.  Use the most recent occurrence in which you had forgone specialist care due to mandatory deductible payments when answering the next questions. |
| --- |
| **An example question**  Consider your most recent situation in which you did not use specialist care. Assume that a new system has been introduced which applies an annual cap set at €385 and that you have not used health care subject to cost-sharing yet.  Which option do you prefer?   1. Directly pay 10% of the price per visit or treatment to the hospital. 2. Pay 100 euros per visit or treatment to the health insurer (billed afterwards). 3. Choose not to use specialist care (forgo health care, no cost-sharing payments).   *Explanation*  Option (A) implies that you pay 10% of the price per visit or treatment and have to pay this directly to the hospital. Option (B) implies that you pay 100 euros per visit or treatment, but you are billed for this payment by the health insurer afterwards (i.e. you use specialist care and receive the bill later at an unknown moment). Option (C) implies that you choose not to use specialist care and you do not have to pay. |

| Use the most recent occurrence in which you had forgone specialist care due to mandatory deductible payments when answering the next questions. Please indicate which of the possible options you prefer in each situation: |
| --- |
| 1. Situation 1*   Consider your most recent situation in which you did not use specialist care. Assume that a new system has been introduced which applies an annual cap set at €385 and that you have not used health care subject to cost-sharing yet.  Which option do you prefer?   1. Pay 7% of the price per visit or treatment to the health insurer (billed afterwards). 2. Directly pay 200 euros per visit or treatment to the hospital. 3. Choose not to use specialist care (forgo health care, no cost-sharing payments). |
| 1. Situation 2*   Consider your most recent situation in which you did not use specialist care. Assume that a new system has been introduced which applies an annual cap set at €385 and that you have not used health care subject to cost-sharing yet.  Which option do you prefer?   1. Directly pay 10% of the price per visit or treatment to the hospital. 2. Pay 100 euros per visit or treatment to the health insurer (billed afterwards). 3. Choose not to use specialist care (forgo health care, no cost-sharing payments). |
| 1. Situation 3*   Consider your most recent situation in which you did not use specialist care. Assume that a new system has been introduced which applies an annual cap set at €385 and that you have not used health care subject to cost-sharing yet.  Which option do you prefer?   1. Directly pay 5% of the price per visit or treatment to the hospital. 2. Pay 140 euros per visit or treatment to the health insurer (billed afterwards). 3. Choose not to use specialist care (forgo health care, no cost-sharing payments). |

| Use the most recent occurrence in which you had forgone specialist care due to mandatory deductible payments when answering the next questions. Please indicate which of the possible options you prefer in each situation: |
| --- |
| 1. Situation 4*   Consider your most recent situation in which you did not use specialist care. Assume that a new system has been introduced which applies an annual cap set at €385 and that you have paid cost-sharing payments equal to half of the annual cap.  Which option do you prefer?   1. Pay 5% of the price per visit or treatment to the health insurer (billed afterwards). 2. Directly pay 140 euros per visit or treatment to the hospital. 3. Choose not to use specialist care (forgo health care, no cost-sharing payments). |
| 1. Situation 5*   Consider your most recent situation in which you did not use specialist care. Assume that a new system has been introduced which applies an annual cap set at €385 and that you have paid cost-sharing payments equal to half of the annual cap.  Which option do you prefer?   1. Pay 200 euros per visit or treatment to the health insurer (billed afterwards). 2. Directly pay 7% of the price per visit or treatment to the hospital. 3. Choose not to use specialist care (forgo health care, no cost-sharing payments). |
| 1. Situation 6*   Consider your most recent situation in which you did not use specialist care. Assume that a new system has been introduced which applies an annual cap set at €385 and that you have paid cost-sharing payments equal to half of the annual cap.  Which option do you prefer?   1. Directly pay 5% of the price per visit or treatment to the hospital. 2. Pay 200 euros per visit or treatment to the health insurer (billed afterwards). 3. Choose not to use specialist care (forgo health care, no cost-sharing payments). |

| Different fixed amounts and percentages were used in the previous questions. Fixed amounts consisted of 100, 140 and 200 euros per visit or treatment. Proportional amounts reflected 5%, 7% or 10% of the price per visit or treatment.  It has been reported that, on average, patients do not know the costs of specialist care. We would like to know more about this.  Use the most recent occurrence in which you had forgone specialist care due to mandatory deductible payments when answering the next questions. |
| --- |
| 1. Do you know the average costs of a visit or treatment?   *If so, enter a rounded amount in euros……* [text box] |
| 1. Do you think that the different fixed amounts and percentages used in the previous questions are: 2. Way too high. 3. Too high. 4. Just right. 5. Too low. 6. Way too low. |
| 1. We would like to know any additional explanations for your previous answers. We would also like to know any other reasons why you had forgone specialist care. If desired, you may also make any other comments regarding forgoing health care.   [text box] |

# Version 1B1 Prescribed Medication – Group: *Utilizing health care*

| You answered not to have forgone health care due to mandatory deductible payments in the past two years.  You have may have used health care covered by the basic health insurance package and recommended by a physician in the past two years. Most health services covered by this package is subject to mandatory deductible payments up to an annual cap of €385. We would like to know more about the effect of these payments on health care use.  *Note: visits to your General Practitioner do not apply here as these visits are not subject to mandatory deductible payments.* |
| --- |
| 1. Have you picked up prescribed medicines from the pharmacy for which you had to pay for them through the mandatory deductible in the past two years? 2. Yes, I have picked up medicines for which I had to pay for them through the mandatory deductible. 3. Yes, I have picked up medicines, but I do not know whether I had to pay for them through the mandatory deductible. 4. Yes, I have picked up medicines, but I did not have to pay for them through the mandatory deductible. 5. No, I did not have to pick up any medicines. 6. I do not know anymore. |
| Use the most recent occurrence in which you had picked up prescribed medicines when answering the next questions.   1. Which type of prescribed medicines have you forgone? 2. Pain relievers. 3. Cholesterol-lowering medication. 4. Stomach acid inhibitors. 5. Anti-hypertensives. 6. Antibiotics / Penicillin. 7. Diuretics. 8. Asthma medication / COPD medication. 9. Eczema medication. 10. Blood thinners. 11. Diabetes medication. 12. Anti-epileptic drugs. 13. Antidepressants. 14. Hay fever medication. 15. Otherwise, namely…… [text box] 16. I do not know anymore. |
| 1. We would like to know about any comments on the previous questions you may have.   [text box] |

| **The current payment system**  If you use health care covered by the mandatory basic health insurance package and subject to the mandatory deductible, you have to pay deductible payments up to 385 euros in 2016.  Prescribed medicines are covered by the mandatory basic health insurance package and you have to pay (part) of their costs depending on any previous payments due to the mandatory deductible. When you fill your prescription at the pharmacy, you generally do not pay the pharmacy directly. Your health insurer pays the pharmacy and sends you a bill for cost-sharing payments afterwards.  The health insurer sends the bill to you afterwards. This may take several weeks to months. If you pick up medicines at the pharmacy, you often do not know much you have to pay as this depends on, for example, on the quantity and price of the medicines.  **New payment system**  Suppose that the government considers a new system that replaces the current system of mandatory deductibles. In this new system, annual cost-sharing payments are capped at €385 and two different types of payments are possible:   - A fixed amount per box of medicines (e.g. €4 per box). - A percentage of the actual price per box of medicines (e.g. 50% of the price).   In this new system you pay either directly to the pharmacy or you are billed afterwards by your health insurer for this payment. You have the option to forgo health care by choosing not to fill the medicines (e.g. if you cannot or do not want to pay for the medicines).  **Following questions**  In the following questions, a new system is described and three options are provided. We would like you to indicate which of these options you prefer the most if the current system of mandatory deductibles is replaced.  Use the most recent occurrence in which you had picked up prescribed medicines when answering these questions. |
| --- |
| **An example question**  Consider your most recent situation in which you picked up prescribed medicines. Assume that a new system has been introduced which applies an annual cap set at €385 and that you have not used health care subject to cost-sharing yet.  Which option do you prefer?   1. Directly pay 2 euros per medicine box to the pharmacy. 2. Pay 70% of the price per medicine box to the health insurer (billed afterwards). 3. Choose not to pick up the medicines (forgo health care, no cost-sharing payments).   *Explanation*  Option (A) implies that you pay 2 euros per box of medicines and have to pay this directly to the pharmacist. Option (B) implies that you pay 70% of the price per medicine box, but you are billed for this payment by the health insurer afterwards (i.e. you pick up the medicines and receive the bill later at an unknown moment). Option (C) implies that you choose not to pick up the medicines and you do not have to pay. |

| Use the most recent occurrence in which you had picked up prescribed medicines when answering next questions. Please indicate which of the possible options you prefer in each situation: |
| --- |
| 1. Situation 1*   Consider your most recent situation in which you picked up prescribed medicines. Assume that a new system has been introduced which applies an annual cap set at €385 and that you have not used health care subject to cost-sharing yet.  Which option do you prefer?   1. Pay 2 euros per medicine box to the health insurer (billed afterwards). 2. Directly pay 70% of the price per medicine box to the pharmacy. 3. Choose not to pick up the medicines (forgo health care, no cost-sharing payments). |
| 1. Situation 2*   Consider your most recent situation in which you picked up prescribed medicines. Assume that a new system has been introduced which applies an annual cap set at €385 and that you have not used health care subject to cost-sharing yet.  Which option do you prefer?   1. Directly pay 4 euros per medicine box to the pharmacy. 2. Pay 40% of the price per medicine box to the health insurer (billed afterwards). 3. Choose not to pick up the medicines (forgo health care, no cost-sharing payments). |
| 1. Situation 3*   Consider your most recent situation in which you picked up prescribed medicines. Assume that a new system has been introduced which applies an annual cap set at €385 and that you have not used health care subject to cost-sharing yet.  Which option do you prefer?   1. Directly pay 70% of the price per medicine box to the pharmacy. 2. Pay 4 euros per medicine box to the health insurer (billed afterwards). 3. Choose not to pick up the medicines (forgo health care, no cost-sharing payments). |

| Use the most recent occurrence in which you had picked up prescribed medicines when answering the next questions. Please indicate which of the possible options you prefer in each situation: |
| --- |
| 1. Situation 4*   Consider your most recent situation in which you picked up prescribed medicines. Assume that a new system has been introduced which applies an annual cap set at €385 and that you have paid cost-sharing payments equal to half of the annual cap.  Which option do you prefer?   1. Directly pay 50% of the price per medicine box to the pharmacy. 2. Pay 7 euros per medicine box to the health insurer (billed afterwards). 3. Choose not to pick up the medicines (forgo health care, no cost-sharing payments). |
| 1. Situation 5*   Consider your most recent situation in which you picked up prescribed medicines. Assume that a new system has been introduced which applies an annual cap set at €385 and that you have paid cost-sharing payments equal to half of the annual cap.  Which option do you prefer?   1. Pay 70% of the price per medicine box the health insurer (billed afterwards). 2. Directly pay 2 euros per medicine box to the pharmacy. 3. Choose not to pick up the medicines (forgo health care, no cost-sharing payments). |
| 1. Situation 6*   Consider your most recent situation in which you picked up prescribed medicines. Assume that a new system has been introduced which applies an annual cap set at €385 and that you have paid cost-sharing payments equal to half of the annual cap.  Which option do you prefer?   1. Directly pay 2 euros per medicine box to the pharmacy. 2. Pay 50% of the price per medicine box to the health insurer (billed afterwards). 3. Choose not to pick up the medicines (forgo health care, no cost-sharing payments). |

| Different fixed amounts and percentages were used in the previous questions. Fixed amounts consisted of 2, 4 and 7 euros per medicine box. Proportional amounts reflected 40%, 50% or 70% of the price per medicine box.  It has been reported that, on average, patients do not know the costs of their medications. We would like to know more about this.  Use the most recent occurrence in which you had picked up prescribed medicines when answering next questions. |
| --- |
| 1. Do you know the average costs of your medicines?   *If so, enter a rounded amount in euros……* [text box] |
| 1. Do you think that the different fixed amounts and percentages used in the previous questions are: 2. Way too high. 3. Too high. 4. Just right. 5. Too low. 6. Way too low. |
| 1. We would like to know any additional explanations for your previous answers.   [text box] |

# Version 1B2 Diagnostic tests – Group: *Utilizing health care*

| 1. Have you undergone diagnostic tests for which you had to pay for them through the mandatory deductible in the past two years? 2. Yes, I have undergone diagnostic tests for which I had to pay for them through the mandatory deductible. 3. Yes, I have undergone diagnostic tests, but I do not know whether I had to pay for them through the mandatory deductible. 4. Yes, I have undergone diagnostic tests, but I did not have to pay for them through the mandatory deductible. 5. No, I did not have undergone diagnostic tests. 6. I do not know anymore. |
| --- |
| Use the most recent occurrence in which you had undergone diagnostic tests when answering the next questions.   1. Which type of diagnostic tests have you undergone? 2. Blood tests. 3. Urinary or faeces tests. 4. X-rays, a CAT or MRI scan, ultrasound test. 5. Cycling test or lung function test. 6. Otherwise, namely…… [text box] 7. I do not know anymore. |
| 1. We would like to know about any comments on the previous questions you may have.   [text box] |

| **The current payment system**  If you use health care covered by the mandatory basic health insurance package and subject to the mandatory deductible, you have to pay deductible payments up to 385 euros in 2016.  Diagnostic tests are covered by the mandatory basic health insurance package and you have to pay (part) of their costs depending on any previous payments due to the mandatory deductible. When you undergo diagnostic tests at a hospital, you generally do not pay the hospital directly. Your health insurer pays the hospital and sends you a bill for cost-sharing payments afterwards.  The health insurer sends the bill to you afterwards. This may take several weeks to months. If you undergo diagnostic tests at a hospital, you often do not know much you have to pay as this depends on, for example, on the type and price of the examination.  **New payment system**  Suppose that the government considers a new system that replaces the current system of mandatory deductibles. In this new system, annual cost-sharing payments are capped at €385 and two different types of payments are possible:   - A fixed amount per examination (e.g. €50 per blood test examination or per CAT scan). - A percentage of the actual price per examination (e.g. 30% of the price).   In this new system you pay either directly to the hospital or you are billed afterwards by your health insurer for this payment. You have the option to forgo health care by choosing not to undergo the diagnostic tests (e.g. if you cannot or do not want to pay for the examination).  **Following questions**  In the following questions, a new system is described and three options are provided. We would like you to indicate which of these options you prefer the most if the current system of mandatory deductibles is replaced.  Use the most recent occurrence in which you had undergone diagnostic tests when answering the next questions. |
| --- |
| **An example question**  Consider your most recent situation in which you had undergone diagnostic tests. Assume that a new system has been introduced which applies an annual cap set at €385 and that you have not used health care subject to cost-sharing yet.  Which option do you prefer?   1. Directly pay 40 euros per examination to the hospital. 2. Pay 30% of the examination’s price to the health insurer (billed afterwards). 3. Choose not to undergo the examination (forgo health care, no cost-sharing payments).   *Explanation*  Option (A) implies that you pay 40 euros per examination and have to pay this directly to the hospital. Option (B) implies that you pay 30% of the price per examination, but you are billed for this payment by the health insurer afterwards (i.e. you undergo the examination and receive the bill later at an unknown moment). Option (C) implies that you choose not to undergo the examination and you do not have to pay. |

| Use the most recent occurrence in which you had undergone diagnostic tests when answering the next questions. Please indicate which of the possible options you prefer in each situation: |
| --- |
| 1. Situation 1*   Consider your most recent situation in which you had undergone diagnostic tests. Assume that a new system has been introduced which applies an annual cap set at €385 and that you have not used health care subject to cost-sharing yet.  Which option do you prefer?   1. Pay 15% of the examination’s price to the health insurer (billed afterwards). 2. Directly pay 50 euros per examination to the hospital. 3. Choose not to undergo the examination (forgo health care, no cost-sharing payments). |
| 1. Situation 2*   Consider your most recent situation in which you had undergone diagnostic tests. Assume that a new system has been introduced which applies an annual cap set at €385 and that you have not used health care subject to cost-sharing yet.  Which option do you prefer?   1. Pay 80 euros per examination to the health insurer (billed afterwards). 2. Directly pay 20% of the examination’s price to the hospital. 3. Choose not to undergo the examination (forgo health care, no cost-sharing payments). |
| 1. Situation 3*   Consider your most recent situation in which you had undergone diagnostic tests. Assume that a new system has been introduced which applies an annual cap set at €385 and that you have not used health care subject to cost-sharing yet.  Which option do you prefer?   1. Directly pay 15% of the examination’s price to the hospital. 2. Pay 80 euros per examination to the health insurer (billed afterwards). 3. Choose not to undergo the examination (forgo health care, no cost-sharing payments). |

| Use the most recent occurrence in which you had undergone diagnostic tests when answering the next questions. Please indicate which of the possible options you prefer in each situation: |
| --- |
| 1. Situation 4*   Consider your most recent situation in which you had undergone diagnostic tests. Assume that a new system has been introduced which applies an annual cap set at €385 and that you have paid cost-sharing payments equal to half of the annual cap.  Which option do you prefer?   1. Pay 20% of the examination’s price to the health insurer (billed afterwards). 2. Directly pay 80 euros per examination to the hospital. 3. Choose not to undergo the examination (forgo health care, no cost-sharing payments). |
| 1. Situation 5*   Consider your most recent situation in which you had undergone diagnostic tests. Assume that a new system has been introduced which applies an annual cap set at €385 and that you have paid cost-sharing payments equal to half of the annual cap.  Which option do you prefer?   1. Directly pay 30% of the examination’s price to the hospital. 2. Pay 40 euros per examination to the health insurer (billed afterwards). 3. Choose not to undergo the examination (forgo health care, no cost-sharing payments). |
| 1. Situation 6*   Consider your most recent situation in which you had undergone diagnostic tests. Assume that a new system has been introduced which applies an annual cap set at €385 and that you have paid cost-sharing payments equal to half of the annual cap.  Which option do you prefer?   1. Directly pay 15% of the examination’s price to the hospital. 2. Pay 50 euros per examination to the health insurer (billed afterwards). 3. Choose not to undergo the examination (forgo health care, no cost-sharing payments). |

| Different fixed amounts and percentages were used in the previous questions. Fixed amounts consisted of 40, 50 and 80 euros per examination. Proportional amounts reflected 15%, 20% or 30% of the examination’s price.  It has been reported that, on average, patients do not know the costs of their diagnostic tests. We would like to know more about this.  Use the most recent occurrence in which you had undergone diagnostic tests when answering the next questions. |
| --- |
| 1. Do you know the average costs of your diagnostic tests?   *If so, enter a rounded amount in euros……* [text box] |
| 1. Do you think that the different fixed amounts and percentages used in the previous questions are: 2. Way too high. 3. Too high. 4. Just right. 5. Too low. 6. Way too low. |
| 1. We would like to know any additional explanations for your previous answers.   [text box] |

# Version 1B3 Specialist care – Group: *Utilizing health care*

| 1. Have you used visits to or treatment by the medical specialists in the hospital (i.e. specialist care) which you had to pay for them through the mandatory deductible in the past two years? 2. Yes, I have used specialist care for which I had to pay for them through the mandatory deductible. 3. Yes, I have used specialist care, but I do not know whether I had to pay for them through the mandatory deductible. 4. Yes, I have used specialist care, but I did not have to pay for them through the mandatory deductible. 5. No, I did not have used specialist care. 6. I do not know anymore. |
| --- |
| Use the most recent occurrence in which you had used specialist care when answering the next questions.   1. For which specialism have your forgone specialist care? *[dropdown menu]* 2. Cardiology. 3. Dental surgery. 4. Dermatology. 5. ENT medicine. 6. Ophthalmology. 7. Emergency Room. 8. Gastrointestinal medicine. 9. General surgery. 10. Gynaecology. 11. Haematology. 12. Internal medicine. 13. Nephrology. 14. Neurology. 15. Orthopaedics. 16. Pulmonology. 17. Radiotherapy. 18. Rehabilitation medicine. 19. Rheumatology. 20. Otherwise, namely…… [text box] 21. No, I do not know that. |
| 1. We would like to know about any comments on the previous questions you may have.   [text box] |

| **The current payment system**  If you use health care covered by the mandatory basic health insurance package and subject to the mandatory deductible, you have to pay deductible payments up to 385 euros in 2016.  Specialist care is covered by the mandatory basic health insurance package and you have to pay (part) of its costs depending on any previous payments due to the mandatory deductible. When you use specialist care at a hospital, you generally do not pay the hospital directly. Your health insurer pays the hospital and sends you a bill for cost-sharing payments afterwards.  The health insurer sends the bill to you afterwards. This may take several weeks to months. If you use specialist care at a hospital, you often do not know much you have to pay as this depends on, for example, on the diagnosis, the type and price of the specialist care.  **New payment system**  Suppose that the government considers a new system that replaces the current system of mandatory deductibles. In this new system, annual cost-sharing payments are capped at €385 and two different types of payments are possible:   - A fixed amount per visit or treatment (e.g. €140 per visit or treatment). - A percentage of the actual price per visit or treatment (e.g. 7% of the price).   In this new system you pay either directly to the hospital or you are billed afterwards by your health insurer for this payment. You have the option to forgo health care by choosing not to use specialist care (e.g. if you cannot or do not want to pay for the specialist care).  **Following questions**  In the following questions, a new system is described and three options are provided. We would like you to indicate which of these options you prefer the most if the current system of mandatory deductibles is replaced.  Use the most recent occurrence in which you had used specialist care when answering the next questions. |
| --- |
| **An example question**  Consider your most recent situation in which you had used specialist care. Assume that a new system has been introduced which applies an annual cap set at €385 and that you have not used health care subject to cost-sharing yet.  Which option do you prefer?   1. Directly pay 10% of the price per visit or treatment to the hospital. 2. Pay 100 euros per visit or treatment to the health insurer (billed afterwards). 3. Choose not to use specialist care (forgo health care, no cost-sharing payments).   *Explanation*  Option (A) implies that you pay 10% of the price per visit or treatment and have to pay this directly to the hospital. Option (B) implies that you pay 100 euros per visit or treatment, but you are billed for this payment by the health insurer afterwards (i.e. you use specialist care and receive the bill later at an unknown moment). Option (C) implies that you choose not to use specialist care and you do not have to pay. |

| Use the most recent occurrence in which you had used specialist care when answering the next questions. Please indicate which of the possible options you prefer in each situation: |
| --- |
| 1. Situation 1*   Consider your most recent situation in which you had used specialist care. Assume that a new system has been introduced which applies an annual cap set at €385 and that you have not used health care subject to cost-sharing yet.  Which option do you prefer?   1. Pay 5% of the price per visit or treatment to the health insurer (billed afterwards). 2. Directly pay 140 euros per visit or treatment to the hospital. 3. Choose not to use specialist care (forgo health care, no cost-sharing payments). |
| 1. Situation 2*   Consider your most recent situation in which you had used specialist care. Assume that a new system has been introduced which applies an annual cap set at €385 and that you have not used health care subject to cost-sharing yet.  Which option do you prefer?   1. Pay 200 euros per visit or treatment to the health insurer (billed afterwards). 2. Directly pay 7% of the price per visit or treatment to the hospital. 3. Choose not to use specialist care (forgo health care, no cost-sharing payments). |
| 1. Situation 3*   Consider your most recent situation in which you had used specialist care. Assume that a new system has been introduced which applies an annual cap set at €385 and that you have not used health care subject to cost-sharing yet.  Which option do you prefer?   1. Directly pay 5% of the price per visit or treatment to the hospital. 2. Pay 200 euros per visit or treatment to the health insurer (billed afterwards). 3. Choose not to use specialist care (forgo health care, no cost-sharing payments). |

| Use the most recent occurrence in which you had used specialist care when answering the next questions. Please indicate which of the possible options you prefer in each situation: |
| --- |
| 1. Situation 4*   Consider your most recent situation in which you had used specialist care. Assume that a new system has been introduced which applies an annual cap set at €385 and that you have paid cost-sharing payments equal to half of the annual cap.  Which option do you prefer?   1. Pay 7% of the price per visit or treatment to the health insurer (billed afterwards). 2. Directly pay 200 euros per visit or treatment to the hospital. 3. Choose not to use specialist care (forgo health care, no cost-sharing payments). |
| 1. Situation 5*   Consider your most recent situation in which you had used specialist care. Assume that a new system has been introduced which applies an annual cap set at €385 and that you have paid cost-sharing payments equal to half of the annual cap.  Which option do you prefer?   1. Directly pay 10% of the price per visit or treatment to the hospital. 2. Pay 100 euros per visit or treatment to the health insurer (billed afterwards). 3. Choose not to use specialist care (forgo health care, no cost-sharing payments). |
| 1. Situation 6*   Consider your most recent situation in which you had used specialist care. Assume that a new system has been introduced which applies an annual cap set at €385 and that you have paid cost-sharing payments equal to half of the annual cap.  Which option do you prefer?   1. Directly pay 5% of the price per visit or treatment to the hospital. 2. Pay 140 euros per visit or treatment to the health insurer (billed afterwards). 3. Choose not to use specialist care (forgo health care, no cost-sharing payments). |

| Different fixed amounts and percentages were used in the previous questions. Fixed amounts consisted of 100, 140 and 200 euros per visit or treatment. Proportional amounts reflected 5%, 7% or 10% of the price per visit or treatment.  It has been reported that, on average, patients do not know the costs of specialist care. We would like to know more about this.  Use the most recent occurrence in which you had used specialist care when answering the next questions. |
| --- |
| 1. Do you know the average costs of a visit or treatment?   *If so, enter a rounded amount in euros……* [text box] |
| 1. Do you think that the different fixed amounts and percentages used in the previous questions are: 2. Way too high. 3. Too high. 4. Just right. 5. Too low. 6. Way too low. |
| 1. We would like to know any additional explanations for your previous answers.   [text box] |

# Demographics questions

| We would like to assess whether subgroups are more likely to forgo health care. For example, people with a lower income may forgo health care more frequently than those with a higher income. Therefore, the following questions concern your financial situation, age, gender, health and residence.  *Note that your answers in this survey are completely anonymous. Data will be used at group level and cannot be traced back to individuals.* |
| --- |
| Health status can be measured in terms of five dimensions. Please indicate the statement in each of the five dimensions that best describes your health today . |
| *Mobility*   1. Which statement best describes your health today? 2. I have no problems in walking about. 3. I have slight problems in walking about. 4. I have moderate problems in walking about. 5. I have severe problems in walking about. 6. I am unable to walking about. |
| *Self-care*   1. Which statement best describes your health today? 2. I have no problems washing or dressing myself. 3. I have slight problems washing or dressing myself. 4. I have moderate problems washing or dressing myself. 5. I have severe problems washing or dressing myself. 6. I am unable to wash or dress myself. |
| *Usual activities (e.g. work, study, housework, family or leisure activities)*   1. Which statement best describes your health today? 2. I have no problems doing my usual activities. 3. I have slight problems doing my usual activities. 4. I have moderate problems doing my usual activities. 5. I have severe problems doing my usual activities. 6. I am unable to do my usual activities. |
| *Pain and discomfort*   1. Which statement best describes your health today? 2. I have no pain or discomfort. 3. I have slight pain or discomfort. 4. I have moderate pain or discomfort. 5. I have severe pain or discomfort. 6. I have extreme pain or discomfort. |
| *Anxiety and depression*   1. Which statement best describes your health today? 2. I am not anxious or depressed. 3. I am slightly anxious or depressed. 4. I am moderately anxious or depressed. 5. I am severely anxious or depressed. 6. I am extremely anxious or depressed. |

| 1. How would you describe your health today? 2. Very poor. 3. Poor. 4. Moderate. 5. Good. 6. Very good. |
| --- |

| *Sense of mastery*  Please indicate the statement in each of the following questions best describes you today. |
| --- |
| 1. I have little control over the things that happen to me. 2. Strongly disagree. 3. Disagree. 4. Neither agree nor disagree. 5. Agree. 6. Strongly agree. |
| 1. There is really no way I can solve some of the problems I have. 2. Strongly disagree. 3. Disagree. 4. Neither agree nor disagree.. 5. Agree. 6. Strongly agree. |
| 1. There is little I can do to change many of the important things in my life. 2. Strongly disagree. 3. Disagree. 4. Neither agree nor disagree. 5. Agree. 6. Strongly agree. |
| 1. I often feel helpless in dealing with the problems of life. 2. Strongly disagree. 3. Disagree. 4. Neither agree nor disagree. 5. Agree. 6. Strongly agree. |
| 1. Sometimes I feel that I’m being pushed around in life. 2. Strongly disagree. 3. Disagree. 4. Neither agree nor disagree.. 5. Agree. 6. Strongly agree. |
| 1. I can do just about anything I really set my mind to. 2. Strongly disagree. 3. Disagree. 4. Neither agree nor disagree. 5. Agree. 6. Strongly agree. |
| 1. What happens to me in the future mostly depends on me. 2. Strongly disagree. 3. Disagree. 4. Neither agree nor disagree. 5. Agree. 6. Strongly agree. |

| *Household situation*   1. What is your current household situation? 2. Living alone, never been married. 3. Married or living together. 4. Divorced, not living together. 5. Widow/widower, not living together. 6. Living with parents. 7. Otherwise, namely…… [text box] |
| --- |
| 1. Do you have children living at home? *If so, how many children?* 2. Yes, namely…… [text box] 3. No. |

| *Financial situation*   1. What is your (net) household-income per month? *This is the amount that you receive per month on your bank account.* 2. Less than €1000,-. 3. Between €1001,- and €2000,-. 4. Between €2001,- and €3000,-. 5. Between €3001,- and €4000,-. 6. More than Between €4000,-. 7. I do not want to say / I do not know. |
| --- |
| 1. What is your financial situation like right now? 2. I am accumulating debt. 3. I am using my/our savings. 4. I can just get by. 5. I save some money. 6. I save a lot of money. |

| *General questions*   1. Are you a man or a woman? 2. Man 3. Woman |
| --- |
| 1. What is your birthdate? *dropdown menu*   *[Day – Month – Year]* |
| 1. Do you have one or more chronic conditions? 2. None. 3. Asthma/COPD. 4. Rheumatic condition. 5. Intestinal condition. 6. Diabetes. 7. Cardiovascular disease. 8. Cancer. 9. Physical disability. 10. Gastrointestinal condition. 11. Mental health problems. 12. Mental disability. 13. Other, namely…… [text box] |
| 1. What is the highest grade or level of education that you have completed? 2. No education completed. 3. Primary education (elementary school). 4. Lower vocational education. 5. Preparatory or short secondary vocational education. 6. Secondary general education. 7. Senior secondary vocational education. 8. Senior general secondary education or university preparatory education. 9. Higher vocational Education. 10. Academic education. 11. Post-academic education (PhD) 12. Otherwise, namely…… [text box] |
