# Supplementary material for: The effect of cost-sharing design characteristics on use of health care recommended by the treating physician; a discrete choice experiment
Source: BMC Health Serv Res. 2018 Oct 20;18:797. doi: 10.1186/s12913-018-3598-4 (PMC6195970; doi:10.1186/s12913-018-3598-4)
Supplement: Supplementary file 4 — Additional output. This file contains an overview of reasons for exclusion of respondents and a selection of response on open-ended questions. (DOCX 26 kb) [file 12913_2018_3598_MOESM4_ESM.docx]

# Additional file 2, Additional output

**Excluded respondents**

In total 842 respondents were excluded for various reasons (table 5). Most excluded respondents referred to health services not covered by the basic health insurance package (n=260; 30.9%), or did not disclose the type of health service recommended by their physician (n=311; 37.0%).

Table 7, Excluded respondents and exclusion criteria

| Reasons | Number of respondents (%) | Forgoing healthcare | Utilizing healthcare | Unknown |
| --- | --- | --- | --- | --- |
|  |  |  |  |  |
| *General* |  |  |  |  |
| Incomplete questionnaire due to technical errors in survey tool | 38 (4.7%) |  |  | 38 |
| Respondents under the age of 18 years | 2 (.2%) |  |  | 2 |
|  |  |  |  |  |
| *Health services* |  |  |  |  |
| Covered health services and subject to cost-sharing but was not directed to any choice sets | 88 (10.8%) | 88 |  |  |
| Covered health services, exempt from cost-sharing | 1 (0.1%) | 1 |  |  |
|  |  |  |  |  |
| Not covered health services | 260 (32.0%) | 242 | 18 |  |
| Not-disclosed by respondents | 311 (38.3%) | 14 | 297 |  |
| Health service misclassified by respondents resulting in completion of wrong choice sets | 6 (0.7%) | 6 |  |  |
|  |  |  |  |  |
| *Methodological* |  |  |  |  |
| Left-to-right bias+ | 107 (13.2%) | 21 | 86 |  |
|  |  |  |  |  |
| *Total* | 813 | 372 | 401 | 40 |

+Respondents were also excluded if they consistently chose the first alternative in each choice set. This indicated that they did not trade off any attributes.

**Open-ended questions**

Table 8, Short selection of response indicating limited price-awareness among respondents

| The questionnaire is complex to complete, especially due to the nominal and proportional fees. I have learned that we (healthcare users) know too little or do not want to know about costs of care [respondent 4446]. |
| --- |
| I cannot compare the nominal and proportional fees since I do not know the costs of my medication…. [respondent 5753] |
| It remains a gamble what you will pay if cost-sharing depends on unknown prices…. That’s why I consistently choose the alternative in which payments have nominal / known fees… [respondent 873] |
| It is difficult to determine actual cost-sharing payments since you do not know the exact costs of diagnostic tests or specialist care. You only receive these bills afterwards… [respondent 8898] |
| I do not know the costs of care [8654] |
| It is difficult since I have no idea the costs of diagnostic tests. These prices should be made public [respondent 1541] |
| I do not know the costs of medication [respondent 6096] |
| I do not know the prices of my medication [respondent 7422] |
| On multiple occasions, I have received a bill pay deductibles due to a medical treatment that took place up to one year prior. That is unacceptable. Moreover, it is difficult since the costs of care are not unclear in advance. Both the GP and hospital are not able to inform me on costs [respondent 7422]. |
